# Hemispheric asymmetry in high-energy phosphate consumption during sleep-deprivation is balanced by creatine

**DOI:** 10.3389/fnins.2025.1515761

**Published:** 2025-05-30

**Authors:** Ali Gordji-Nejad, Andreas Matusch, Sophie Kleedörfer, Harshal Jayeshkumar Patel, Alexander Drzezga, David Elmenhorst, Ferdinand Binkofski, Andreas Bauer

**Affiliations:** ^1^Institute of Neuroscience and Medicine (INM-2), Molecular Organization of the Brain, Forschungszentrum Jülich, Jülich, Germany; ^2^Division of Clinical Cognitive Sciences, Department of Neurology Uniklinik RWTH Aachen, RWTH Aachen University Hospital, Aachen, Germany; ^3^Department of Nuclear Medicine, Faculty of Medicine and University Hospital Cologne, University of Cologne, Cologne, Germany; ^4^German Center for Neurodegenerative Diseases (DZNE), Bonn-Cologne, Germany; ^5^Institute of Neuroscience and Medicine (INM-4), Medical Imaging Physics, Forschungszentrum Jülich, Jülich, Germany

**Keywords:** hemispheric asymmetry, sleep deprivation, creatine, high energy phosphate, ^31^P-magnetic resonance spectroscopy, cognitive performance

## Abstract

The human brain exhibits asymmetric hemispheric activity at night; this plays a crucial role in cognitive impairment from sleep deprivation. Although there have been many investigations on this topic, there are no studies on hemispheric differences in the consumption of high-energy phosphates (HEP). We present here a new data analysis of our previously published study in which subjects were measured for changes in high-energy phosphate (HEP), tCr/tNAA, and Glu/TNAA during subacute sleep deprivation (21 h) and cognitive tests. In our new analysis, we investigated differences and asymmetries in the metabolic consumption of both hemispheres. Comprehensive per-individual voxel-wise interhemispheric comparisons at all time points and conditions showed a greater decrease from baseline of ATP in the right than in the left hemisphere. Partial volume correction yielded an apparent higher decline of PCr/Pi in gray versus white matter. We also investigated whether creatine supplementation, which has been shown to prevent cognitive impairment during sleep deprivation, affected this hemispheric asymmetry. In a second session, the subjects took a high single dose of creatine monohydrate suspension (0.35 g/kg) after baseline measurements. Creatine balanced the sleep deprivation-induced asymmetry to a higher degree in the left hemisphere, which was due to an increase in PCr/Pi and decrease in ATP. Our results confirm—via the observed decrease in ATP level—a night-active right hemisphere. Creatine administration balanced this asymmetry.

## 1 Introduction

Brain lateralization is a crucial factor in neuronal and cognitive functions (Mutha, 2012; Duboc, 2015; Reber, 2017; Güntürkün, 2020). In this context, sleep and sleep deprivation appear to influence asymmetric hemispheric activity, which in turn affects cognitive abilities (Casagrande, 2010). The regions affected, such as the prefrontal, parietal, and temporal cortices, influence alertness, the vigilance system, and working memory (Posner, 1990; Sturm, 1999; Cabeza, 2000; Raz and Buhle, 2006; Bartolomeo, 2019). Numerous studies observed increased neuronal activity in the cortical networks of the right compared to the left hemisphere at night and during sleep deprivation (SD). This finding was accentuated during attention-focused working memory tasks. fMRI investigations showed an increased activation of the right prefrontal cortex and parietal region during the divided attention task after SD (Drummond, 2001).

Since these studies were performed using modalities such as EEG or fMRI, there is currently a lack of studies using magnetic resonance spectroscopy (MRS) to detect changes in high-energy phosphates (HEP) such as ATP or PCr.

On the other hand, various studies have reported improved cognitive performance after a long-term high-creatine diet (Rae, 2003; McMorris, 2006; Rae and Broer, 2015; Roschel, 2021; Forbes, 2022; Candow, 2023; Sandkühler, 2023; Machado, 2024). The substance counteracts the metabolic changes caused by sleep deprivation (Lyoo, 2003; Pan and Takahashi, 2007; Cook, 2011; Dworak, 2017). Our recent study showed that creatine reverses the metabolic changes in HEP and cognitive deterioration caused by sleep deprivation (Gordji-Nejad, 2024).

Based on all these findings, we hypothesize that sleep deprivation also causes hemispheric asymmetry in HEP consumption. If so, creatine supplementation would be expected to influence it. As the right hemisphere is involved in the control of attention, alertness, and vigilance, this could explain the prevention of the deterioration in cognitive performance caused by sleep deprivation. Consequently, it could provide clues for the future use of creatine to clarify whether it is primarily effective at night or during sleep deprivation, as it causes changes in neuronal networks and hemispheric activity during this time.

In this study, hemispheric changes in high-energy phosphate (HEP), tCr/tNAA, and Glu/TNAA were analyzed in the same cohort described in our previous study (Gordji-Nejad, 2024). Subjects underwent subacute sleep deprivation (21 h) and passed four runs, each comprising cognitive tests and MRS in a quiescent awake eyes-open state. In one session, subjects received a high single dose of creatine monohydrate suspension (0.35 g/kg) after the baseline scan; in the other session on another day, the same subjects received a placebo. Our results show a higher metabolic consumption in the night-active right hemisphere due to a decrease in ATP, which is compensated for after creatine administration.

## 2 Materials and methods

More detailed information is given in and some key features are repeated here (Gordji-Nejad, 2024).

### 2.1 Participants

This randomized, prospective, double-blind, balanced cross-over design study was conducted at the University Hospital RWTH Aachen, Germany. Fifteen healthy subjects (8 women, 7 men, aged 23 ± 2 years, range 20–28, 13 right-handed) participated. All were free of neurological diseases, psychiatric and sleep disorders, and alcohol or drug abuse. The study complied with the Declaration of Helsinki in its latest version and was approved by the local ethics committee of the Medical Faculty of RWTH Aachen University. A declaration of consent was obtained from all subjects.

### 2.2 Experimental procedure

**Figure 1:**
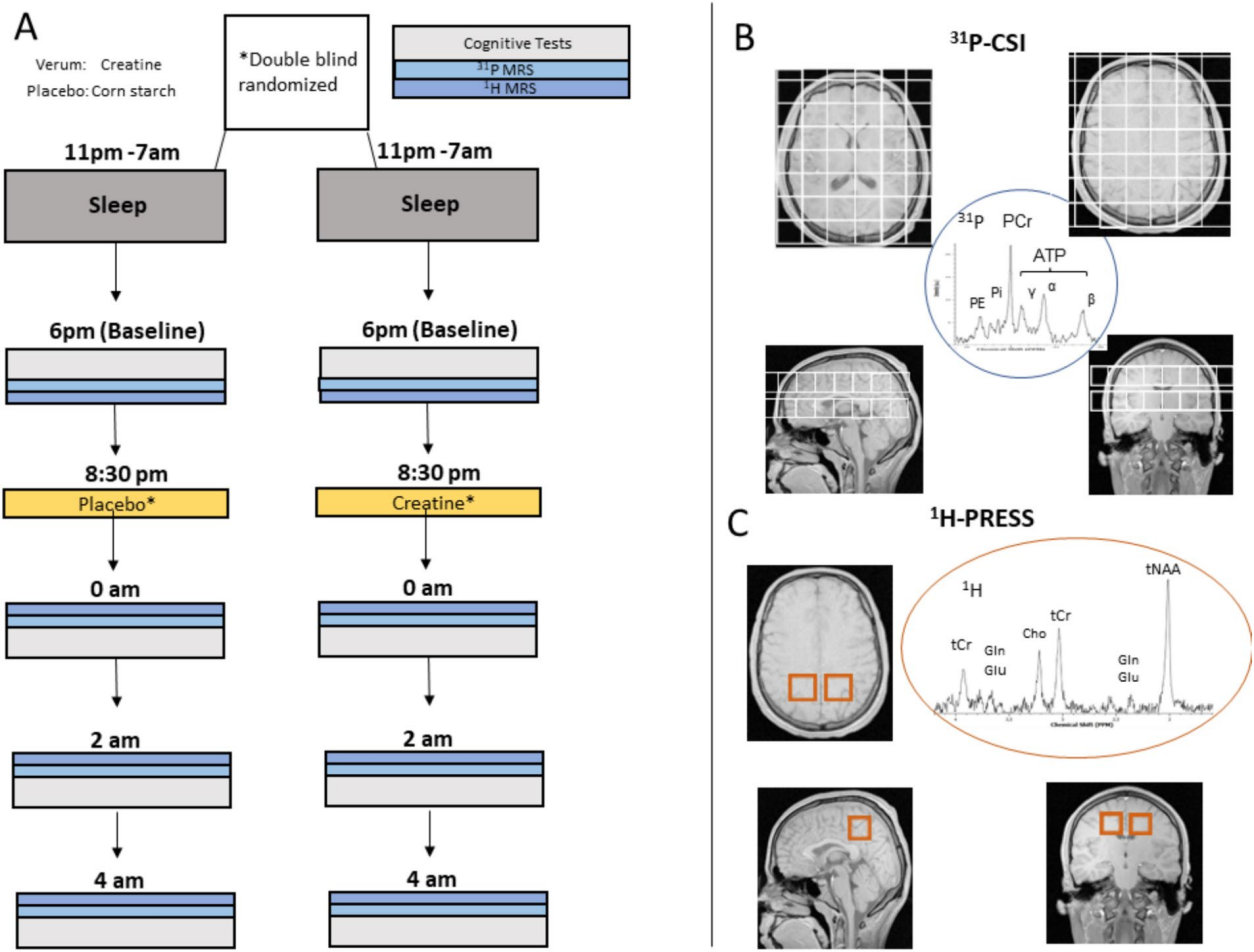
**(A)** Study design: in one session, the subjects were administered creatine at 8:30 p.m. and in another they were administered a placebo. The order of the sessions was random. The interval between the two sessions was at least 5 days. In each session, cognitive and metabolic parameters were acquired in four runs. The baseline started at 6 p.m., the other at 0 a.m., 2 a.m., and 4 a.m. Each session included two ^31^P-MRS and three ^1^H-MRS measurements, followed by fatigue scores, psychomotor vigilance tests (PVT), and other cognitive tasks (Cog.Test) and lasted 1 h and 35 min. **(B)** Positioning of two 8 × 8 ^31^P-MRS CSI grids. The isotropic voxel size was (25 mm)^3^ in the coronal, transversal, and sagittal view. The signals exploited were of ^31^P-MRS PCr, ATP-*β*, and Pi. **(C)** Positioning of two single voxel ^1^H-MR-spectroscopy (PRESS) voxels each (25 mm)^3^. The signals of total creatine (tCr) and glutamate (Glu) were evaluated.

Each subject was measured on two nights (Figure 1A) with a minimum interval of 5 days (max. 27, mean 10 ± 6 days) in between. On one night, 0.35 g/Kg creatine (creatinemonohydrate, AlzChem, Trostberg), on the other, 0.35 g/Kg placebo (corn starch, Caelo GmbH) was administered. The recommended daily dose is 3–5 grams. The rationale of administering a high dose was to ensure that, in case of increased uptake, sufficient extracellular creatine was available. A further reason was that, in addition to the fact that the uptake in the neurons is low compared to the muscles, the subjects respond differently to creatine. The aim was to maximize the expected uptake even in non-responders, where the expected uptake is lower. Throughout both sessions, subjects were not allowed to sleep, were permanently supervised, were optically monitored inside and outside the MR scanner, and were spoken to whenever initial signs of falling asleep occurred. The camera and pulse oximeter were used to monitor the pulse and movements, which are particularly important during sleep deprivation. Karolinska Sleepiness Scores (KSS, ranged from 1 to 10) and fatigue score (FAT, ranged from 1 to 20) were assessed before and after each run. FAT was the 10-item inverted version of the Samn & Perelli Fatigue score.

The baseline measurement started at 6 p.m. Thereafter, subjects were orally administered with 0.35 g/Kg creatine or 0.35 g/Kg placebo at 8:30 p.m. and measured in subsequent runs at 0 p.m., 2 am, and 4 am. Each run comprised two ^31^P-MRS measurements, three ^1^H-MRS measurements, and a neuropsychological test battery outside the scanner (Figure 1A).

#### 2.2.1 Cognitive tasks

The test battery consisted of a Psychomotor Vigilance test (PVT), Word Memory Test (WMT), forward memory digit span test (SPAN), spatial N-Back (3-Back), and multiple-choice language, logic, and numeric tasks (Gordji-Nejad, 2024).

In the PVT reaction time test, subjects had to press a button as fast as possible when a green LED appeared. In the word memory test (WMT), 22 word pairs were presented on a laptop and in SPAN 12 random single-digit numbers were presented. During the recall phase, participants were asked to type the second word of each word pair for WMT and the correct order of the numbers for SPAN. In the spatial triple-N algorithm, a sequence of 3 × 3 matrices was presented. Here, a button would have to be pressed if the position of a square matched the third previous one. The cognitive multiple-choice tests included the categories language (21 tasks), logic (17 tasks), and numeric (8 tasks). The tests were divided into two series, A and B, each with four batteries for the two measurement nights. The order of the two series was randomized and balanced across the subjects.

#### 2.2.2 MRI/MRS measurement

Measurements were performed on a 3.0 Tesla Magnetom Prisma scanner (Siemens) using a dual-tuned ^31^P-^1^H head coil from Rapid Biomedical (Würzburg, Germany). Anatomical reference images were acquired using T1-weighted 2D flash sequences.

##### 2.2.2.1 MRS sequences

Two 2D chemical shift imaging (CSI) ^31^P-MRS sequences were applied to acquire axial grids of 8 × 8 voxels (25 mm)^3^. One grid was placed in a midplane centered on the thalamus and the other, measured immediately after, above the corpus callosum (Figure 1B). Subsequently, ^1^H spectroscopy was performed on two (25 mm)3 medial parietal single voxels using a PRESS (WS) sequence (Figure 1C).

##### 2.2.2.2 MRS data analysis

MRS data were analyzed with TARQUIN 4.3.10. All data underwent a Fourier transformation as well as zero and first-order phase correction. From ^31^P-CSI, spectra of 2 × 9 voxels and both ^1^H-PRESS voxels containing the entire brain tissue were evaluated. Data were fitted as a linear combination of the metabolic basis set, including PCr, ATP-*β*, Pi, PE, TCHo, GPC, GPE, tCr, tNAA, Cho, Glx, and GABA. To evaluate changes within each metabolite, the ratio of the metabolite to the total phosphorus signal, including the sum of the signal integral of PCr, ATP-β, Pi, PE, and TCHo (declared as 31P), was selected.

### 2.3 Statistics and evaluation

Due to the longitudinal design, the within subject version of the two-tailed *T*-test was used to compare the mean values of the MRS values and cognitive performance.

Changes in cognitive parameters and in metabolic parameters in each voxel at 0 a.m., 2 a.m., or 4 a.m. were calculated versus baseline and averaged throughout individuals. Right–left differences of changes were calculated thereafter (Tables 1, 2). Changes versus placebo were calculated as the difference between the changes in the creatine and placebo session (Supplementary material 2.3).

**Table 1:** Mean within-subject differences of PCr/Pi, ATP-ß, tCr/TNAA, and Glu/TNAA of right versus left hemisphere throughout the middle and upper ^31^P-CSI slices and ^1^H single voxels (SVS) after placebo and creatine at each single time point 6 p.m., 0 p.m., 2 a.m., and 4 a.m.

Middle slice		PCr/Pi								ATP-ß							
Voxel (l vs. r)	Anatomical label	6 p.m.		0 a.m.		2 a.m.		4 a.m.		6 p.m.		0 a.m.		2 a.m.		4 a.m.	
		Placebo	Creatine	Placebo	Creatine	Placebo	Creatine	Placebo	Creatine	Placebo	Creatine	Placebo	Creatine	Placebo	Creatine	Placebo	Creatine
R4 (C3–C6)	Anterior insula	−3%	−1%	−3%	−1%	−3%	1%	5%	7%	3%	5%	5%	3%	1%	5%	0%	0%
R5 (C3–C6)	Temporal transversal	−7%	−5%	−0%	−10%*	−11%**	2%	2%	5%	8%*	3%	3%	6%	−3%	1%	2%	3%
R6 (C3–C6)	Post. sup temporal	−7%	−1%	−3%	−19%**	−6%	3%	3%	3%	5%	−1%	1%	1%	−1%	4%	3%	0%
R6 (C4–C5)	Corpus callosum	−3%	−2%	−1%	−3%**	−2%	2%	1%	−1%	1%	−2%	−0%	2%	−3%	2%	−1%	0%
R7 (C3–C6)	Occipito-temporal	−10%	1%	−2%	−18%*	−5%	2%	−0%	7%	4%	−4%	1%	3%	−2%	0%	3%	0%
R3 (C4–C5)	Anterior cingulum	0%	1%	−3%	−2%	−1%	−3%	2%	3%*	−1%	2%	−3%	2%	4%**	0%	1%	−1%
R4 (C4–C5)	Caudate-putamen	−2%	−1%	−1%	−1%	−1%	−3%	4%*	5%*	1%	−2%	0%	3%	0%	1%	−2%	−1%
R5 (C4–C5)	Thalamo-capsular	−4%	−3%	1%	−3%	2%	1%	2%	−0%	3%	−5%*	0%	−1%	−2%	0%	0%	−4%**
R7 (C4–C5)	Occipito-medial	−1%	2%	−1%	−5%	−4%	3%	4%	1%	2%	−2%	−2%	−3%	−3%	−3%	−3%*	−3%**

Upper slice		6 p.m.		0 a.m.		2 a.m.		4 a.m.		6 p.m.		0 a.m.		2 a.m.		4 a.m.	
		Placebo	Creatine	Placebo	Creatine	Placebo	Creatine	Placebo	Creatine	Placebo	Creatine	Placebo	Creatine	Placebo	Creatine	Placebo	Creatine
R4 (C3–C6)	Lateral premotor	−3%	−3%	−3%	4%	−12%	1%	5%	−6%	12%	9%	0%	9%*	6%	17%	−8%	−9%
R5 (C3–C6)	Motor	−4%	1%	−3%	−2%	−2%	−4%	9%	−5%	10%*	5%	−3%	5%	5%	11%*	−9%	0%
R6 (C3–C6)	Ant. later. Parietal	−1%	1%	−3%	−8%*	1%	3%	3%	−2%	4%	4%	1%	−6%	1%	11%*	−7%	0%
R6 (C4–C5)	Medial central	1%	0%	−2%	−4%**	−3%	2%	−1%	−1%	0%	0%	3%	4%	3%	1%	−4%	4%
R7 (C3–C6)	Post. lateral parietal	10%	−4%	4%	−13%*	3%	−1%	−10%	1%	3%	0%	−3%	−8%	5%	15%	−7%	5%
R3 (C4–C5)	Anterior F1	−3%	1%	3%	3%	−3%	2%	1%	−3%	0%	4%	2%	6%*	−6%	8%	1%	8%
R4 (C4–C5)	Posterior F1	−4%	0%	1%	1%	−4%	1%	4%	−2%	3%	4%*	1%	1%	5%	8%	−2%	2%
R5 (C4–C5)	Medial premotor Post. lateral parietal	−1%	−3%	2%	−1%	−3%	−1%	3%	3%	4%*	4%	−2%	2%	1%	8%**	−5%*	−3%
R7 (C4–C5)	Precuneus	2%	0%	−1%	−3%	2%	0%	−3%	−4%	0%	1%	0%	−5%	2%	0%	−5%	−2%

Anatomical label		tCr/TNAA								Glu/TNAA							
		6 p.m.		0 a.m.		2 a.m.		4 a.m.		6 p.m.		0 a.m.		2 a.m.		4 a.m.	
		Placebo	Creatine	Placebo	Creatine	Placebo	Creatine	Placebo	Creatine	Placebo	Creatine	Placebo	Creatine	Placebo	Creatine	Placebo	Creatine
r-l	Ant. med. Parietal	0%	4%*	−2%	3%	1%	0%	2%	0%	7%	17%	3%	3%	−5%	21%	−5%	8%

**p = values of *p* ≤ 0.005; *p = values of 0.0063 ≤ *p* ≤ 0.05, that did not survive Bonferroni correction.

**Table 2:** Mean within-subject percentage change of PCr/Pi, ATP-ß, tCr/TNAA, and Glu/TNAA versus baseline (6 p.m.), left hemispheric value subtracted from right hemispheric value ((riS_T_/riS_B_ − 1) − (leS_T_/leS_B_−1)); S_T_, S_B_, signal at timepoint T and baseline B; r, right; l, left, within the middle and upper ^31^P-CSI slices and ^1^H single voxels (SVS) after placebo and creatine at 0 p.m., 2 a.m., and 4 a.m.

Middle slice		PCr/Pi						ATP-ß					
Voxel (l vs. r)	Anatomical label	0 a.m.		2 a.m.		4 a.m.		0 a.m.		2 a.m.		4 a.m.	
		Placebo	Creatine	Placebo	Creatine	Placebo	Creatine	Placebo	Creatine	Placebo	Creatine	Placebo	Creatine
R4 (C3–C6)	Anterior insula	1%	−1%	−1%	0%	7%	7%	0%	−5%*	−1%	−2%	−3%	−6%
R5 (C3–C6)	Temporal transversal	9%	−5%	−4%	4%	7%	13%	−6%	−1%	−12%*	−2%	−5%	−3%
R6 (C3–C6)	Post. sup temporal	1%	−17%*	0%	−2%	10%	3%	−4%	−1%	−6%	3%	−2%	0%
R6 (C4–C5)	Corpus callosum	2%	−1%	1%	0%	4%	3%	−2%	4%	−4%*	4%	−3%	2%
R7 (C6–C3)	Occipito-temporal	5%	−18%*	0%	−4%	5%	6%	−4%	3%	−5%	4%	−1%	1%
R3 (C4–C5)	Anterior cingulum	−1%	−3%	−3%	2%	2%	1%	0%	−3%	5%*	−4%	2%	−3%
R4 (C4–C5)	Caudate-putamen	1%	−1%	−2%	4%	5%	6%*	−1%	1%	−2%	2%	−3%	−4%*
R5 (C4–C5)	Thalamo-capsular	4%	0%	4%	1%	5%	3%	−2%	5%*	−4%	4%	−2%	1%
R7 (C4–C5)	Occipito-medial	0%	−7%	3%	−4%	3%	1%	−4%*	−1%	−5%	−1%	−6%*	−1%

Upper slice		0 a.m.		2 a.m.		4 a.m.		0 a.m.		2 a.m.		4 a.m.	
		Placebo	Creatine	Placebo	Creatine	Placebo	Creatine	Placebo	Creatine	Placebo	Creatine	Placebo	Creatine
R4 (C3–C6)	Lateral premotor	1%	6%	−9%	1%	6%	−4%	−11%	−2%	−3%	2%	−19%*	−15%
R5 (C3–C6)	Motor	−2%	−2%	4%	−5%	15%*	−4%	−14%	0%	−5%	2%	−18%*	−6%
R6 (C3–C6)	Ant. later. Parietal	−1%	−7%	4%	2%	5%	−2%	−4%	−11%	−3%	7%	−12%	−5%
R6 (C4–C5)	Medial central	−3%	−2%	−3%	2%	−2%	0%	3%	2%	4%	1%	−3%	2%
R7 (C3–C6)	Post. lateral parietal	−4%	−11%	−5%	−1%	−14%	2%	−6%	−7%	−1%	6%	−6%	−2%
R3 (C4–C5)	Anterior F1	5%	0%	−2%	1%	4%	−4%	3%	4%	−4%	1%	5%	2%
R4 (C4–C5)	Posterior F1	5%	1%	−1%	1%	8%*	−2%	−3%	−2%	3%	3%	−4%	−3%
R5 (C4–C5)	Med. premotor	3%	2%	−1%	2%	4%	6%	−7%*	−2%	−2%	2%	−10%**	−7%
R7 (C4–C5)	Precuneus	−2%	−2%	1%	−1%	−4%	−3%	0%	−6%	1%	1%	6%	−5%

Anatomical label		tCr/TNAA						Glu/TNAA					
		0 a.m.		2 a.m.		4 a.m.		0 a.m.		2 a.m.		4 a.m.	
		Placebo	Creatine	Placebo	Creatine	Placebo	Creatine	Placebo	Creatine	Placebo	Creatine	Placebo	Creatine
r-l	Ant. med. Parietal	−3%	−1%	5%	−4%	2%	−1%	−5%	−17%	0%	12%	−18%	0%

**p = values of *p* ≤ 0.005; *p = values of 0.0063 ≤ *p* ≤ 0.05, that did not survive Bonferroni correction.

Due to the limited sample size, possible false Type error II had to be considered. Due to the multiple tests (k = 8, 2 sessions of 4 runs each), the significance thresholds were adjusted to *α* = 0.05/8 = 0.0063 to compare individual voxels using the Bonferroni approach. False discovery rate (FDR) consideration Type error I due to multiple testing of 9 bilateral voxel wise comparison for 4 condition resulted in α = 0.05/36 = 0.0014. The correlations between the changes in the metabolic parameters and the cognitive values were analyzed by calculating the Pearson correlation coefficient (r).

Hemispheric difference was defined as the respective parameter of a left hemispheric voxel minus the right homologue. For each voxel and each measurement, a variance-weighted statistic was applied regarding the baseline-related and placebo-verum difference. Details are given in the Supplementary material.

#### 2.3.1 Reliability and reproducibility

The fraction of white matter (WM), gray matter (GM), and cerebrospinal fluid (CSF) was measured for each voxel using SPM8 and the respective anatomical MPRAGE-MRI scan. This allowed the estimation of the contribution of variance in head positioning to the variation of MRS signal between measurement runs (Supplementary material 2.4, 2.5). In addition, differences in baseline values from session one and session two and the variability between and within subjects across all regions and conditions were compared. Multiple phantom measurements on different nights were performed to calculate the signal drifts from baseline to each time point.

#### 2.3.2 Partial volume correction

A further methodological question was whether correction of ^31^P-MRS-signals and signal-ratios for the part attributable to gray matter or white matter could reduce between subject variability and, thus, statistical robustness of the cohort. If *frac. GM* and *frac. WM* are the fractions for each voxel determined by segmentation, the total signal, e.g., *ΔPCr* is:


(1) $$\text{ΔPCr}=\mathrm{\Delta PC}{r_{\mathrm{GM}}}^{*}(\text{frac}.\mathrm{GM})+\mathrm{\Delta PC}{r_{\mathrm{WM}}}^{*}(\text{frac}.\mathrm{WM})$$

whereby *ΔPCr_GM_* and *ΔPCr_WM_* denote the signal attributable to gray matter and white matter, respectively. The contribution of the CSF compartment is neglected in this approach as it was reported to amount only to 0.0007 times the total signal for both PCr and Pi.

This correction approach would enable an estimate of the error induced by shifts in head positioning between sessions and measurement runs. However, the biological validity of such a correction has limitations, as additional assumptions are required, namely a fixed ratio of signal originating from gray matter and white matter, respectively, constant over the entire brain and over all subjects. Here, we assumed a fixed ratio of *PCr*_GM_/*PCr*_WM_ = 1.2, of *Pi*_GM_/*Pi*_WM_ = 1, based on the literature given in the methods section of the Supplementary material (Zhu, 2004; Dudley, 2014; Ruhm, 2021). The partial volume corrected signals of Eq. [1] are then:


$$\begin{array}{l} \Delta {\left(\mathrm{PCr}/\mathrm{Pi}\right)}_{\mathrm{GM}}=\Delta (\mathrm{PCr}/\mathrm{Pi})/[(\text{frac}.\mathrm{GM})+(\text{frac}.\mathrm{WM})/1.2]\;\text{and}\;\\ \Delta {\left(\mathrm{PCr}/\mathrm{Pi}\right)}_{\mathrm{WM}}=\Delta (\mathrm{PCr}/\mathrm{Pi})/[1.2^{*}\;(\text{frac}.\mathrm{GM})+(\text{frac}.\mathrm{WM})] \end{array}$$

## 3 Results

All 15 subjects completed the study. The sub-total sleep deprivation was effective, significantly impacting on established parameters, which are discussed in more detail below. None of the subjects fell asleep during scans and sessions as assessed by monitoring eyes, responsiveness, respiration, and pulse oximetry. Creatine was well tolerated; no gastric discomfort was signalized. Comprehensive information about the entire results can be found in our previously published paper (Gordji-Nejad, 2024).

Regarding ^31^P MRS including PCr/Pi and ATP-ß, the middle grid centered onto the thalamus included the anterior insular, anterior cingulum, striatal, temporal, occipital, corpus callosum, and thalamic region. The upper grid above the corpus callosum included the premotor, anterior F1, posterior F1, motor, medial central, precuneus, and parietal region. Regarding ^1^H MRS including tCr/NAA and Glu/tNAA, two voxels included the left and right medial posterior parietal region. Due to the short correlation time of the nucleic dipole–dipole moments in the cytosol, it can be assumed that the observed ^31^P-MRS and ^1^H signals are restricted to this location (Luby-Phelps, 2000).

**Figure 2:**
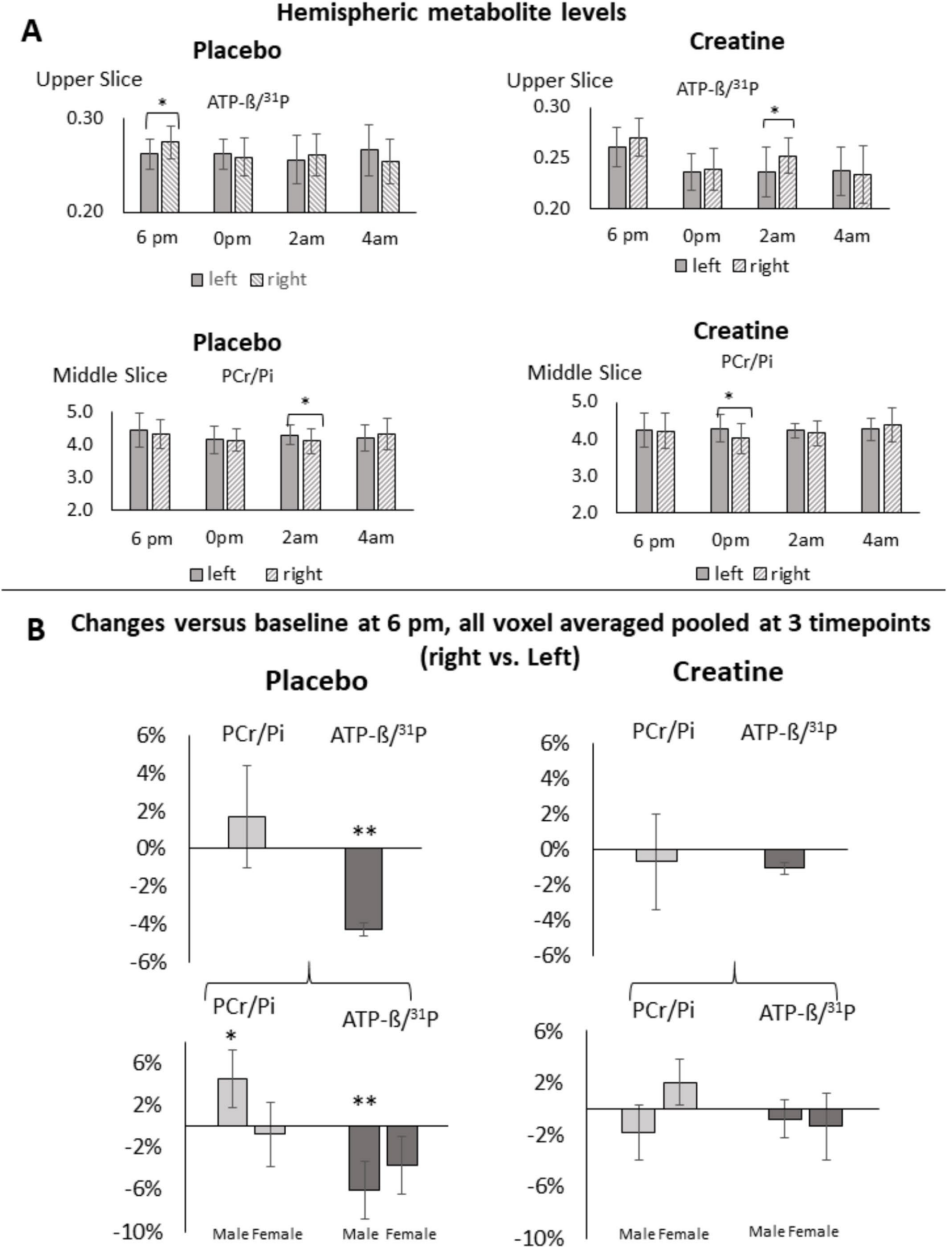
**(A)** PCr/Pi and ATP-ß/^31^P levels in averaged middle and upper grid voxels of the left and right hemisphere after placebo or creatine treatment at measurement time points 6 p.m., 0 p.m., 2 a.m., and 4 a.m. * = *p* ≤ 0.05 indicates significant differences. **(B)** Changes referenced to baseline (6 p.m.) in ATP-ß/^31^P in averaged voxels after placebo or creatine treatment at measurement time points 0 p.m., 2 a.m., and 4 a.m. * = *p* ≤ 0.05 indicates significant differences, ** = *p* ≤ 0.005 indicates significant differences withstanding the Bonferroni correction.

**Figure 3:**
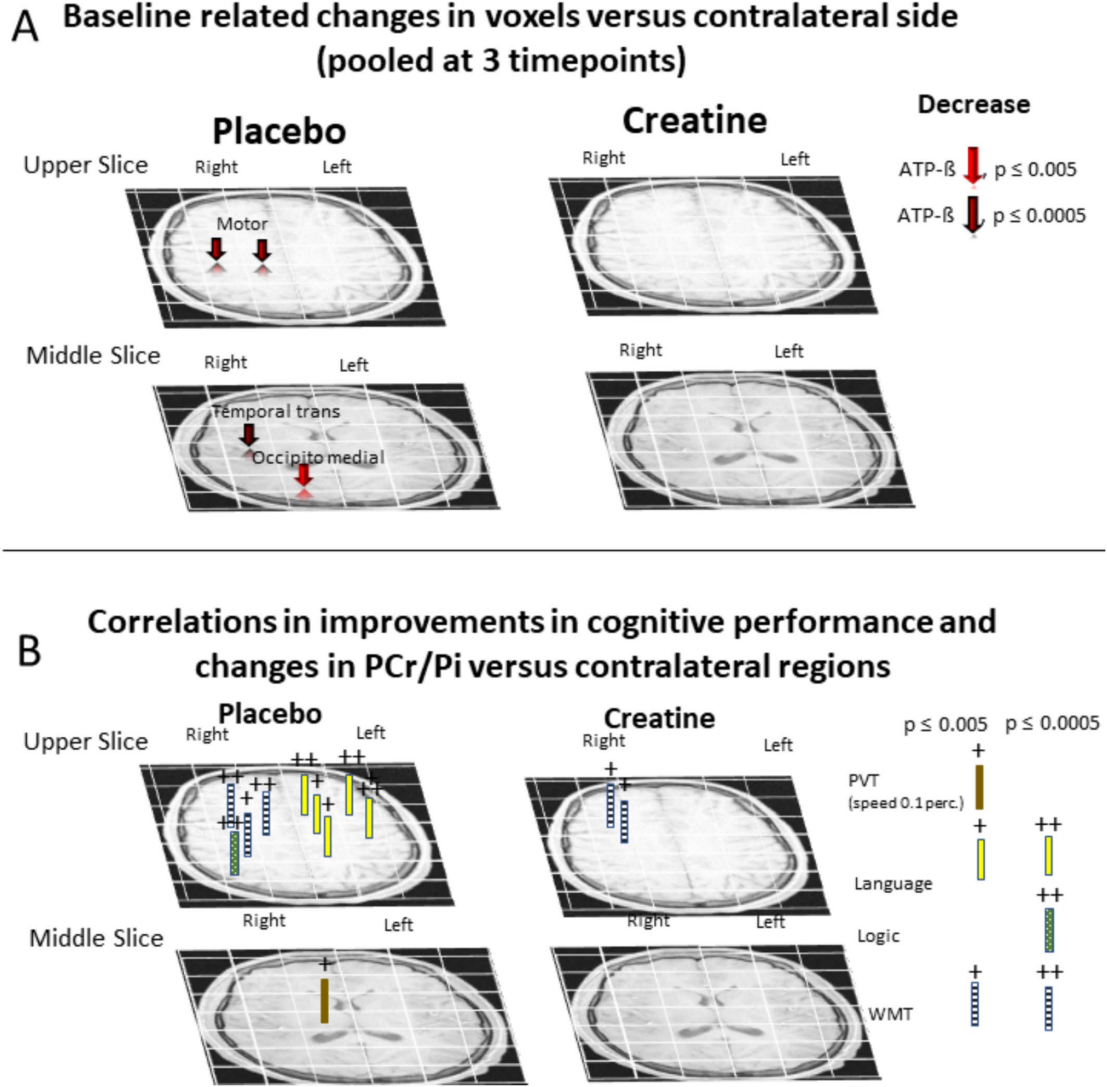
**(A)** Changes versus baseline (6 p.m.) in middle and upper grid voxels in PCr/Pi (large arrows) and ATP-ß/^31^P (small arrows) versus contralateral hemisphere after placebo or creatine administration pooled at 0 a.m., 2 a.m., and 4 a.m. Significance levels are color-coded. The illustration is in radiological orientation (the right side of the brain is on the left side of the viewer). Changes withstanding the Bonferroni correction are colored in red, those withstanding the Benjamini Hochberg correction are in dark red. **(B)** Positive correlations in baseline-related increases of PCr/PI in voxel versus contralateral regions and improvements in cognitive performance when pooled at three time points (0 p.m., 2 a.m., and 4 a.m.), withstanding the Bonferroni corrections.

**Figure 4:**
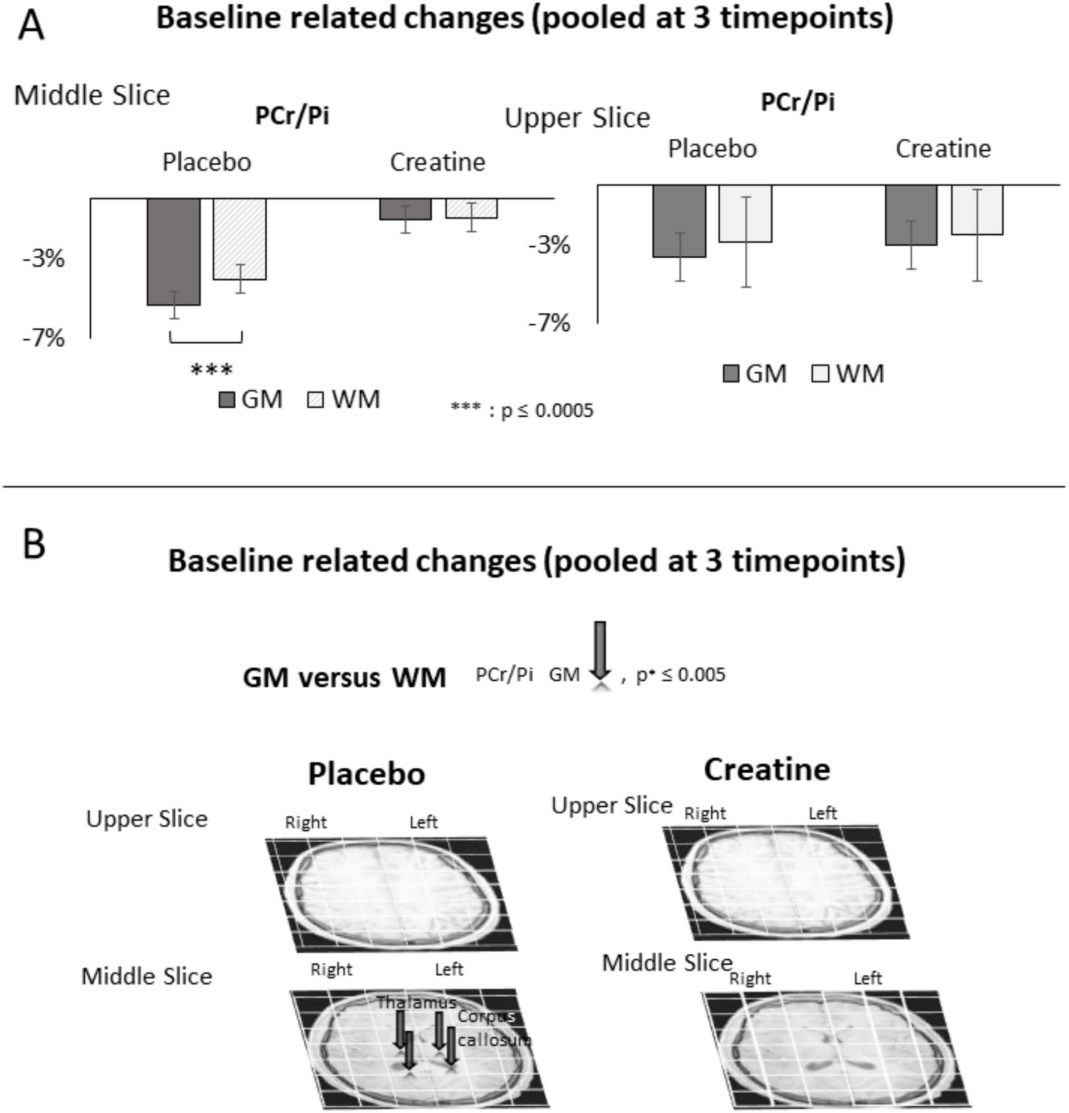
Baseline-related (6 p.m.) changes in white matter (WM) and gray matter (GM) in PCr/Pi after placebo or creatine administration in **(A)** averaged middle and upper grids and in **(B)** gray versus white matter in voxels pooled at three time points (0 a.m., 2 a.m., and 4 a.m.). PCr/Pi (gray arrow) decreases significantly more than white matter, withstanding the Bonferroni correction.

Relative intra-individual hemispheric differences in changes of PCr/Pi, ATP-ß, tCr/NAA, and Glu/tNAA at each single time point versus baseline (6 p.m.) and pooled at 0 p.m., 2 am, and 4 am averages across all subjects after administration of placebo or creatine are shown in Tables 1–3, Figures 2, 3A, and Supplementary Figures S1, S2. The differences in changes between creatine and placebo, constituting the net response, are given in Table 4 and Supplementary Figure 3C. Correlations between changes of PCr/Pi and ATP-ß level in the left versus right hemisphere and cognitive performance are given in Supplementary Tables S1a–d and Figure 3B. Changes of PCr/Pi in gray versus white matter after placebo or creatine administration are shown in Table 5 and Figure 4.

**Table 3:** Mean within-subject percentage change of PCr/Pi and ATP-ß versus baseline (6 p.m.), left versus right hemispheric value within all averaged voxels of the middle and upper ^31^P-CSI slices after placebo and creatine pooled at 0 p.m., 2 a.m., and 4 a.m.

Session	Parameter	Left versus right	
Placebo	Δ*PCr/Pi*	Female	0.8%
		Male	−4.5%*
	Δ*ATP-ß/^31^P*	Female	3.7%
		Male	6.1%**
Verum	Δ*PCr/Pi*	Female	−2%
		Male	1.8%
	Δ*ATP-ß/^31^P*	Female	1.4%
		Male	0.8%

**p = values of *p* ≤ 0.005; *p = values of 0.0063 ≤ *p* ≤ 0.05, that did not survive Bonferroni correction.

**Table 4:** Right–left difference at each time point (0 a.m., 2 a.m., 4 a.m.) referenced to the difference at baseline (6 p.m.) of PCr/Pi, ATP-ß, tCr/TNAA, and Glu/TNAA ((ri*S*_T_−le*S*_T_)/(ri*S*_B_−le*S*_B_)−1; S_T_, S_B_, signal at timepoint T and baseline B; ri, right; le, left) throughout the middle and upper ^31^P-CSI slices and ^1^H single voxels (SVS) of creatine versus placebo at 0 p.m., 2 a.m., and 4 a.m.

Middle Slice		PCr/Pi			ATP-ß		
Voxel (l vs. r)	Anatomical label	0 a.m.	2 a.m.	4 a.m.	0 a.m.	2 a.m.	4 a.m.
R4 (C3–C6)	Anterior insula	1%	−1%	7%	−5%	1%	−4%
R5 (C3–C6)	Temporal transversal	9%	−4%	7%	5%	11%*	3%
R6 (C3–C6)	Post. sup temporal	1%	0%	10%	3%	10%	2%
R6 (C4–C5)	Corpus callosum	2%	1%	4%	6%	9%**	5%
R7 (C3–C6)	Occipito-temporal	5%	0%	5%	11%	8%	−1%
R3 (C4–C5)	Anterior cingulum	−1%	−3%	2%	0%	−9%*	−6%
R4 (C4–C5)	Caudate-putamen	1%	−2%	5%	2%	3%	−1%
R5 (C4–C5)	Thalamo-capsular	4%	4%	5%	5%	8%**	1%
R7 (C4–C5)	Occipito-medial	0%	−3%	3%	5%	6%	6%

Upper slice		0 a.m.	2 a.m.	4 a.m.	0 a.m.	2 a.m.	4 a.m.
R4 (C3–C6)	Lateral premotor	−2%	−1%	−1%	10%	8%	3%
R5 (C3–C6)	Motor	−10%	7%	7%	11%	6%	11%
R6 (C3–C6)	Ant. later. Parietal	−15%	−1%	−5%	−1%	12%	4%
R6 (C4–C5)	Medial central	−3%	−2%	−1%	−2%	−3%	6%
R7 (C3–C6)	Anterior F1	−20%	−5%	4%	1%	9%	7%
R3 (C4–C5)	Posterior F1	−3%	4%	−3%	3%	9%	5%
R4 (C4–C5)	Medial premotor	−3%	4%	1%	0%	1%	1%
R5 (C4–C5)	Post. lateral parietal	−5%	−4%	−3%	4%	5%	4%
R7 (C4–C5)	Precuneus	−7%	−1%	−2%	−4%	0%	1%

Anatomical label		tCr/tNAA			Glu/tNAA		
		0 a.m.	2 a.m.	4 a.m.	0 a.m.	2 a.m.	4 a.m.
r-l	Ant. med. Parietal	0%	−7%	1%	11%	8%	24%

**p = values of *p* ≤ 0.005; *p = values of 0.0063 ≤ *p* ≤ 0.05, that did not survive Bonferroni correction.

**Table 5:** Change of PCr/Pi in gray matter (GM) versus white matter (WM) throughout the middle and upper ^31^P-CSI slices after placebo and creatine at 0 p.m., 2 a.m., and 4 a.m. versus baseline at 6 p.m.

Middle Slice			ΔPCr/Pi (GM-WM)							
Voxel No	Hs	Anatomical label	0 p.m. vs. 6 p.m.		2 a.m. vs. 6 p.m.		4 a.m. vs. 6 p.m.		Pooled at 0 p.m., 2 a.m. and 4 a.m.	
			Placebo	Creatine	Placebo	Creatine	Placebo	Creatine	Placebo	Creatine
R5C3	r	Temporal transversal	−0.01%	−0.1%	−2.0%*	0.2%	0.2%	1.5%	−0.6%	0.6%
R5C6	l		−2.2%	1.6%	−1.1%	−0.3%	−1.4%	0.2%	−1.6%*	0.5%
R6C4	r	Corpus callosum	−1.9%*	−0.8%	−2.4%*	−0.6%	−1.0%	1.0%	−1.8%**	−0.1%
R6C5	l		−2.4%*	0.3%	−3.1%*	−0.2%	−1.5%	0.9%	−2.3%**	0.3%
R3C4	r	Anterior cingulum	−4.0%	−2.6%	−0.2%	−0.1%	−0.6%	−0.4%	−0.6%	−0.4%
R3C5	l		−0.4%	0.04%	0.8%	0.2%	−1.1%	−0.5%	−0.3%	−0.1%
R4C4	r	Caudate-putamen	−1.2%	−0.4%	−1.1%	0.2%	−1.0%	−0.1%	−1.1%	−0.1%
R4C5	l		−1.3%	−0.3%	−0.6%	−0.4%	−2.2%*	−1.1%	−1.4%*	−0.6%
R5C4	r	Thalamo-capsular	−1.5%*	−0.3%	−1.6%	−0.2%	−1.2%	0.2%	−1.4%**	−0.1%
R5C5	l		−2.5%**	−0.1%	−2.4%**	−0.7%	−2.5%**	−0.1%	−2.4%**	−0.3%
R7C4	r	Occipito-medial	−1.0%	−1.6%*	−2.3%*	−1.4%	−0.4%	0.4%	−1.2%*	−0.9%
R7C5	l		−0.6%	0.1%	−1.5%	−0.7%	−0.7%	0.5%	−0.9%	−0.04%

Upper slice			0 p.m. vs. 6 p.m.		2 a.m. vs. 6 p.m.		4 a.m. vs. 6 p.m.		Pooled at 0 p.m., 2 a.m. and 4 a.m.	
			Placebo	Creatine	Placebo	Creatine	Placebo	Creatine	Placebo	Creatine
R4C3	r	Lateral premotor	0.2%	0.1%	−1.7%	−0.2%	0.2%	−0.2%	−0.4%	−0.1%
R4C6	l		−0.6%	−1.6%	0.0%	−2.9%	−1.8%	−0.9%	−0.8%	−1.6%
R5C3	r	Motor	0.1%	−0.6%	−0.5%	−2.1%	0.3%	−2.0%	0.04%	−1.4%*
R5C6	l		−0.2%	−0.5%	−1.6%	−1.8%	−2.8%	−0.5%	−1.3%	−0.9%
R6C3	r	Ant. later. Parietal	−0.5%	−1.3%	−0.7%	−1.4%	−1.8%	−0.9%	−0.8%	−1.0%
R6C6	l		−0.1%	0.2%	−2.0%	−1.7%	−3.0%	−0.2%	−1.4%	−0.5%
R4C4	r	Posterior F1	0.0%	−0.2%	−0.7%	7.4%	0.5%	−0.7%	−0.04%	1.8%
R4C5	l		−1.1%	−0.7%	−0.6%	−2.3%	−1.6%	−0.3%	−1.0%	−0.9%
R5C4	r	Medial premotor	−0.4%	−0.2%	−1.5%	−1.4%	−0.4%	1.4%	−0.6%	0.0%
R5C5	l		−0.7%	−0.9%	−1.2%	−2.0%	−1.2%	−0.5%	−0.8%	−1.0%
R7C3	r	Post. lateral parietal	−0.3%	−0.6%	−0.2%	−1.7%	−4.0%*	0.1%	−1.3%	−0.6%
R7C6	l		0.6%	2.1%	−0.1%	−1.5%	−1.4%	−0.7%	−0.3%	0.1%

**p = values of *p* ≤ 0.005; *p = values of 0.0063 ≤ *p* ≤ 0.05, that did not survive Bonferroni correction.

### 3.1 Hemispheric asymmetry in HEP consumption during SD

No significant hemispheric differences in PCr/Pi level were revealed at baseline (6 p.m.) in the averaged middle and upper grid. At 2 am, a lower level of −4.1 ± 2.1%, p_13_ = 0.04, t_13_ = −2.1 occurred in the right compared to the left hemisphere in the averaged middle grid (Figure 2A). Since these changes were not significant compared to baseline, it can be concluded that SD did not induce significant hemispheric changes in PCr/Pi.

No significant hemispheric differences in ATP-ß/^31^P level occurred at baseline (6 p.m.) in the averaged middle grid. In turn, the upper grid showed a lower ATP-ß/^31^P level (−4.5 ± 2.1%, p_13_ = 0.04, t_13_ = −2.1) in the left compared to the right hemisphere (Figure 2A). Subsequent measurements revealed a decrease in the right compared to left hemisphere versus baseline in all averaged voxels (−4.3 ± 0.4%, *p*_43_ = 0.00086, *t*_43_ = −3.45) when pooled at 3 times withstanding the Bonferroni and Benjamini Hochberg correction (Figure 2B) which was more pronounced in males (−6.1 ± 2.7%, *p*_21_ = 0.003, *t*_21_ = −3.19) than females (−3.7 ± 2.8%, *p*_24_ = 0.07, *t*_24_ = −1.88) (Figure 2B). The decrease was revealed regionally in the temporal transversal (−7.1 ± 1.8%, *p*_43_ = 0.0007, *t*_43_ = −3.63), occipito medial (−4.8 ± 1.6%, *p*_43_ = 0.003, *t*_43_ = −3.13), motor (−13.7 ± 4.2%, *p*_43_ = 0.0003, *t*_43_ = −3.91), and medial premotor (−6.3 ± 1.6%, *p*_43_ = 0.0002, *t*_43_ = −4.01) regions when pooled at three times points, all withstanding the Bonferroni correction (Figure 3A). Regional changes at each time point compared to baseline, including those notwithstanding the Bonferroni and Benjamini Hochberg correction, are shown in Supplementary material 3.1 and Supplementary Figure S3. Our results show that SD induces significant changes in ATP in the right hemisphere.

No significant hemispheric differences were found in tCr/NAA or Glu/tNAA in the voxels located in the medial parietal regions at any time point as well as compared to baseline (6 p.m.) (Supplementary Figure S1; Tables 1, 2).

A positive correlation between higher score versus baseline was found in the language task (pooled at three timepoints) and higher PCr/Pi levels in the left hemisphere in the averaged upper grid (r = 0.52, *p*_43_ = 0.0003, *t*_43_ = 3.97), including the posterior F1 (r = 0.56, *p*_43_ = 0.0001, *t*_13_ = 4.39), medial premotor (r = 0.39, *p*_43_ = 0.007, *t*_43_ = 2.81), lateral premotor (r = 0.59, *p*_43_ = 0.00002, *t*_43_ = 4.79), motor (r = 0.49, *p*_43_ = 0.001, *t*_43_ = 3.64), and medial central (r = 0.42, *p*_43_ = 0.004, *t*_43_ = 3.02) region (Figure 3B; Supplementary Table S1c). In turn, a regional positive correlation in the right hemisphere showed up between higher scores in the WMT task and PCr/Pi levels in medial premotor (r = 0.48, *p*_43_ = 0.0001, *t*_43_ = 3.59), medial central (r = 0.43, *p*_43_ = 0.003, *t*_43_ = 3.09), and motor region (r = 0.49, *p*_43_ = 0.001, *t*_43_ = 3.70). This was also related to higher scores in the logic task in the postero-lateral parietal (r = 0.50, *p*_43_ = 0.0005, *t*_43_ = 3.74) and in PVT in the thalamo-capsular (r = 0.41, *p*_43_ = 0.0005, *t*_43_ = 2.98) region. The results show that, during SD, there is a significant correlation between higher scores in the language-related task and PCr/Pi levels in the left hemisphere and of WMT and PVT in the right hemisphere withstanding the Bonferroni and Benjamini Hochberg correction.

Furthermore, a regional positive correlation in the left hemisphere was found between higher scores in the WMT and higher ATP-ß level in the left occipito-temporal region (r = 0.48, *p*_43_ = 0.001, *t*_43_ = 3.49 and) and in the logic task in the medial premotor (r = 0.50, *p*_43_ = 0.0006, *t*_43_ = 3.72) and posterior F1 (r = 0.48, *p*_43_ = 0.001, *t*_43_ = 3.48) regions (Supplementary Table S1c). Further correlations not withholding the Bonferroni correction are shown in Supplementary Table S1a. The results show a restricted regional correlation between higher scores in WMT and logic tasks and ATP levels in the left hemisphere.

A positive correlation was found between decreased ATP levels in the right hemisphere and higher scores in the KSS in the averaged middle grid (r = 0.49, *p*_43_ = 0.001, *t*_43_ = 3.73). This correlation, along with regional correlations in KSS and FAT presented in Supplementary Table S1e, withstands the Bonferroni and Benjamini Hochberg corrections.

### 3.2 Hemispheric asymmetry in HEP consumption during SD after creatine administration

Also, in the creatine condition, no significant hemispheric differences in PCr/Pi levels were revealed at baseline (6 p.m.) in the averaged middle or upper grid. At 0 p.m., the averaged middle grid showed a significantly lower level in the right compared to the left hemisphere (−5.8 ± 2.5%, *p*_13_ = 0.03, *t*_13_ = −2.47) (Figure 2A). These changes were not significant compared to baseline. Therefore, it can be concluded that creatine administration during SD did not induce significant hemispheric changes in PCr/Pi.

No significant hemispheric differences in ATP-ß/^31^P levels were revealed at baseline (6 p.m.) in the averaged middle and upper grid. At 2 am, a lower level occurred in the left compared to the right hemisphere in the averaged upper grid (−5.8 ± 2.5%, *p*_13_ = 0.03, *t*_13_ = −2.47). The triple contrast of verum versus placebo, SD versus baseline, and right versus left hemisphere in ATP-ß/^31^P was regionally significant at 2 a.m. in the corpus callosum (−8.6 ± 0.6%, *p*_13_ = 0.0003, *t*_13_ = −4.86) and the thalamus (−8.5 ± 0.8%, *p*_13_ = 0.004, *t*_13_ = −3.49), withstanding the Bonferroni correction (Supplementary Figure S3C). Further regional changes notwithstanding the Bonferroni and Benjamini Hochberg correction are shown in Supplementary Figure S3. It can be concluded that creatine administration during SD did not induce significant hemispheric changes in ATP-ß.

No significant hemispheric differences were revealed in tCr/NAA or Glu/tNAA in the voxels located in the medial parietal regions at any timepoint as well as compared to baseline (6 p.m.) (Supplementary Figure S1; Tables 1, 2). A positive correlation was found between higher scores in WMT tasks and changes in PCr/Pi regionally in the right hemisphere in the lateral premotor (r = 0.53, p43 = 0.0002, t43 = 4.06) and motor (r = 0.45, p43 = 0.002, t43 = 3.28) region when values were pooled at three time points. Further correlations, not withstanding the Bonferroni and Benjamini Hochberg corrections, are shown in Supplementary Table S1d.

### 3.3 Findings from partial volume correction

Assignment of the signal to the gray matter and white matter fraction enabled the estimation of the error induced by the spatial shift of the CSI-grid due to positioning of the outcome measure Δ*PCr/Pi* in the range −1.61 to +0.7% in extreme cases and −0.74 to 0.1% in more than 90% of the voxels and pairs of comparison between time points and the baseline in both verum and placebo sessions. The spatial shift was in the range −0.7 mm to 1.3 mm in all directions and all pairs of time points and baseline. Exemplarily, variabilities of individual averages across all 18 voxels, corrected and uncorrected, are indicated in Table 6.

**Table 6:** Between-subject variabilities (σ) of global averages across all 18 voxels before and after partial volume correction.

Session	Parameter	Variance (σ) of signal attributable to		
		Total signal	Gray matter	White matter
Placebo	Δ*PCr/Pi*	1.60%	0.63%	0.20%
Verum		2.80%	0.77%	0.25%

Corrected values for each voxel and comparison are indicated in Table 5 and Supplementary Table S2. It is worth noting that in the placebo condition, a stronger decrease in PCr/Pi in gray than in white matter occurred in the averaged middle grid (PCr/Pi = −1.33 ± 0.5%, *p*_43_ = 6.1× 10^−6^, *t*_43_ = −5.25) when all time points were pooled (Figure 4A). This was most noticeable in the thalamus (left = −2.3 ± 0.4%, *p*_43_ = 1.9× 10^−7^ *t*_43_ = −6.35, right = −1.4 ± 0.4%, *p*_43_ = 0.001 *t*_43_ = −3.68) and corpus callosum (left = −2.2 ± 0.6%, *p*_43_ = 0.0005, *t*_43_ = −3.84, right = −1.7 ± 0.5%, *p*_43_ = 0.001 *t*_13_ = −3.64) (Figure 4B; Table 5).

## 4 Discussion

In this randomized, controlled, double-blinded cross-over trial, we studied the hemispheric differences of response in cerebral PCr/Pi ATP, tCr, Glu levels, and cognitive performance to (i) sub-total sleep deprivation (SD) versus baseline and (ii) to a single high dose of creatine versus placebo during SD. Sleep deprivation induced a higher decrease in ATP in the right than left hemisphere and of PCr/Pi in gray than in white matter. Creatine administration induced a higher response in HEP in the left hemisphere by increasing PCr and decreasing ATP. Our results show an increased HEP consumption in the right hemisphere during SD and cognitive activity, which is outbalanced by the left hemisphere if creatine was administered previously. However, methodological considerations must be taken into account. Although we have calculated the spatial shifts of the CSI grid and the associated impact on signal changes, partial volume effects must be considered. In addition, we assume a fixed ratio of metabolites, which, however, varies regionally. Furthermore, we tried to statistically compensate for the small effect size due to the small number of subjects with longitudinal measurements and multiple runs in each session, which ultimately led to robust significance using statistical threshold tools.

### 4.1 Hemispheric asymmetry in HEP consumption during sleep deprivation

One of the most striking features of sleep deprivation is a reduced alertness and cognitive performance, which is related to the deactivation and activation of neural networks, with the right hemisphere playing a particular role. fMRI investigations showed an increased activation of the right prefrontal cortex and parietal region during the divided attention task after SD (Drummond, 2001). EEG studies reported a shift of activation from left to right hemisphere and a right hemisphere-associated correlation of deterioration after sleep deprivation (Pallesen, 2004; Szelenberger, 2005; Ferreira, 2006).

The right hemisphere is involved in controlling attention, i.e., the vigilance system, which is the first to suffer from sleep deprivation. This is consistent with our findings showing impaired cognitive tests, particularly in the psychomotor vigilance test and the short-term memory test (Gordji-Nejad, 2024), as well as significant metabolic changes in the right hemisphere.

Given that they withstand the statistical thresholds, ATP changes in the right hemisphere are the most robust and reliable findings in this study. Our results show a higher decrease in ATP in the right than in the left hemisphere through all sessions, although most significant at 2 a.m. and 4 a.m. (Figures 2B, 4A,B). This outcome agrees with higher neuronal activity in the right hemisphere during SD reported by others (Drummond and Brown, 2001; Szelenberger, 2005; Lim, 2007).

In turn, a stronger decline in PCr/Pi in the left hemisphere was only observed regionally in the final session at 4 a.m. with less significance. PCr is the most prominent vehicle to transfer energy from the mitochondrial C-space into the cytosol and is also used for transcellular transport (Wallimann, 1992; Braissant, 2010). Furthermore, PCr acts as a buffer for the replenishment of ATP. Hence, in cases of high energy demand, a reduction in PCr/Pi rather than in ATP is to be expected if sufficient enzymatic conversion and supply via diffusion are assured.

As our study does not include day and night timepoints and thus no sampling points, we speculate that a possible explanation of the decrease in ATP might be the influence of circadian effects or sleep pressure acting as an additional factor to higher neuronal activity. Several studies have reported a decrease in ATP in rats in the late hours before sleep (Dworak, 2010) and a reduction in mitochondrial respiration rate and fission-associated ATP production (Simon, 2003; Schmitt, 2018).

In turn, the decrease in PCr/Pi in the left hemisphere shows clear evidence of increased energy consumption due to cognitive performance. Our results show a high number of regional changes in PCr/Pi levels that correlate with cognitive performance scores in the task-related regions (Supplementary Tables S1a,c).

The correlation between PCr/Pi levels in the left hemisphere and language scores (Figure 4C) is consistent with the reported high dependence of left hemisphere neuronal activity on language tasks (Drummond and Brown, 2001; Friederici, 2013). The concerned regions, such as posterior F1, motor, medial, and lateral premotor cortex, are all involved in the Broca and the conductive part of language production (Cabeza, 2000; Friederici, 2013; Shebani, 2022). Furthermore, the dependencies of PVT and logic scores on PCr/Pi levels in the parietal region and in the right hemisphere correspond to the findings of dependence of sustained attention, arousal and intrinsic vigilance, and higher working memory activation (Kinomura, 1996; Portas, 1998; Sturm, 1999; Cabeza, 2000; Drummond and Brown, 2001; Szelenberger, 2005; Raz and Buhle, 2006; Krause, 2017). In contrast to most studies reporting higher activity in the left hemisphere related to word retrieval (Fletcher, 1995; Ries, 2017; Othman, 2020; Shebani, 2022), our WMT results correlate with higher PCr/Pi values in the right hemisphere. Our results are consistent with the recognition analysis by Johnson (2001), which found a higher fMRI activation in the right dorsolateral prefrontal cortex during a verbal learning test focused on recognition and memory processing of verbal material. The correlation of the increased values in KSS and FAT is remarkable, as it indicates an increased state of fatigue with a decrease in ATP in the hemisphere.

Based on our findings, there are further possible explanations that may reveal the reason for a robust and sustained ATP reduction in the right hemisphere compared to the transient regional changes in PCr/Pi. Compensation of ATP depletion by oxidative phosphorylation is indicative of sustained, long-term neuronal stress or fatigue. This is supported by the fact that an increased fatigue state is associated with a decrease in ATP in the right hemisphere, as shown in our results. Given the effects on the right hemisphere, tasks related to alertness and vigilance are therefore expected to be impaired first and most severely. On the other hand, the PCr molecule, which is more mobile and less polar than ATP, is available as an acute buffer that rapidly replenishes the required energy demand via creatine kinase (Wallimann, 1992) and is recovered within a short time range. This is a possible explanation for why no significant depletion of PCr is observed compared to ATP.

Although the changes in our study were revealed in the same hemispheres and mainly the same regions as those reported by others, differences in the modalities have to be considered regarding the conclusion and interpretations. While the fMRI and EEG studies detect in a high time resolution, neuronal activity, or neuronal firing, our MRS data reflect a status of energy turnover of 20 min. Moreover, our measurements took place after the cognitive tests and not at the same time, as in other studies. Hence, our data reflect a long-term energy status which outlasts the measuring time.

Notably, the decline is more balanced in women than in men (Figure 2B), which agrees with findings in the literature (Bourne, 2010; Casagrande, 2010; Hirnstein, 2019). In men, ATP levels decreased stronger in the right hemisphere and PCr/Pi levels were lower, albeit less significantly, in the left hemisphere (Reber, 2017).

Another finding is the stronger decrease of PCr/Pi in the gray than in the white matter, regionally pronounced in the thalamus and corpus callosum. Plante (2014) and Zhang (2024) reported a stronger response to SD and associated recovery sleep in gray matter than in white matter in regions such as the thalamus and corpus callosum. Åkerstedt (2020) and Long (2020) reported a gray matter volume change in the right thalamus after sleep deprivation.

### 4.2 Balanced HEP consumption during sleep deprivation after creatine administration

In contrast to placebo, creatine administration leads to ATP levels being more balanced between hemispheres during the sessions (Figures 2B, 3A). The compensation of hemispheric asymmetry during SD was not due to an increase in ATP in the right hemisphere but to a strong decrease in the left hemisphere (Gordji-Nejad, 2024). We conclude that the increased ATP consumption in the left hemisphere reflects higher utilization during cognitive tasks while PCr/Pi is upregulated in production but diffuses to the sites of consumption only in sub-stoichiometric quantities resulting in a shift of the Cr + ATP ↔ PCr + ADP dynamic equilibrium to the right (Figure 3A). Furthermore, higher creatine uptake, i.e. prevention of decrease in PCr/Pi, appeared to be stronger in the gray matter than in the white matter, which compensated for the sleep deprivation-induced difference. The fact that this was found in regions with higher energy turnover (Gordji-Nejad, 2024) is consistent with the literatur and confirms the correlation of energy demand and consumption.

In view of the findings, the question arises as to whether the effect of creatine is primarily a response to changes in the active right hemisphere during the night or rather to a general high neuronal energy demand. This will have important implications for the future use of creatine. According to the literature and in line with our results, changes in neuronal activity or metabolic changes in the right hemisphere are associated with impairments in alertness, attention, and the vigilance system. If creatine leads to compensation of the hemispheric asymmetry in energy consumption, such as shown in our results, it could be the key factor for the prevention of cognitive impairments and hence most effective at night or during sleep deprivation. This is supported by our finding that the decrease in ATP in the right hemisphere, which was associated with an increased fatigue state, was reversed after creatine administration. On the other hand, if creatine primarily prevents neuronal stress, such as over acidification or oxidative stress (Gordji-Nejad, 2024), it can be concluded that creatine is effective during periods of high neuronal load regardless of the time of day or night. Our data show a stronger correlation between higher scores in task performance and PCr levels in both hemispheres. Creatine showed its greatest effect through increased ATP demand for PCr production which appeared to be in the left hemisphere. This, in turn, suggests that creatine may be most effective in the regions where high energy demand is needed, namely those involved in cognitive performance. This result would support the hypothesis that creatine is effective at any time of the day or night when energy demand is high.

Clarifying these questions opens up the possibility for further research to clarify whether creatine has the potential to reverse metabolic changes or cognitive impairment during generally high neuronal stress or whether it is only effective at night or during sleep deprivation due to specific changes in the neuronal network or activity changes. Both conclusions will have different impacts on the associated use regarding the specific demand. If creatine is only effective at night or during sleep deprivation, it would be useful for professions with unexpectedly long hours such as firefighters, medical workers, or pilots. Otherwise, it would be useful for anyone with a high workload. It would also have a major impact in terms of clinical therapy options. In the first case, creatine would be particularly useful for sleep disorders such as insomnia, whereas in the second case creatine could be useful for all pathologies associated with energy deficits including neurodegenerative diseases.

## 5 Conclusion

Our results show a stronger decrease in ATP in the night-active right than in the left hemisphere during sleep-deprived cognitive activity. Creatine compensated for the hemispheric asymmetry by decreased ATP levels in the left hemisphere. Thus, creatine supplementation did not only improve attention and cognitive performance during prolonged wakefulness but also shifted hemispheric activation pattern within this time. In view of these results and given that creatine has the general property of preventing neuronal stress, future studies could investigate whether it is specifically effective at night or in the case of sleep deprivation or whether it can be used generally in cases of intense cognitive activity.

## Data Availability

The original contributions presented in the study are included in the article/Supplementary material, further inquiries can be directed to the corresponding author.
