# Supplementary material for: Hemispheric asymmetry in high-energy phosphate consumption during sleep-deprivation is balanced by creatine
Source: Front Neurosci. 2025 May 30;19:1515761. doi: 10.3389/fnins.2025.1515761 (PMC12162656; doi:10.3389/fnins.2025.1515761)
Supplement: Supplementary file 1 [file Data_Sheet_1.DOCX]

**Supplements**

To enable a comprehensive overview of experimental data acquired from the same experiment on the same study cohort the reader may view the respective previous paper including the Supplement there, available for free download at https://www.nature.com/articles/s41598-024-54249-9#Sec25

1. **Material and methods**

**2.3 Statistics and evaluations**

The weighted mean hemispheric difference $\bar{{MD}_{hem}}$ of change in peak integral between BL (6 pm) $x_{i}$and another session $y_{i}$ (0 am, 2 am or 2 am) of a given voxel for *n* (n=15) subjects was calculated as difference of change in a right hemispheric voxel (i) $\Delta_{ir}=\left( y_{ir}-x_{ir} \right)$and change in the contralateral left hemispheric homologue voxel $\Delta_{il}=\left( y_{il}-x_{il} \right)$:

(1)

$\bar{{MD}_{hem}}=\frac{\sum_{i=1}^{n} w_{i_{hem\_xy}}\cdot\left[ \Delta_{ir}-\Delta_{il} \right]}{\sum_{i=1}^{n} w_{i_{hem\_xy}}}$ $\bar{\mathrm{MD}}=\frac{\sum_{i=1}^{n} w_{i_{\mathrm{xy}}}\cdot\left( y_{i}-x_{i} \right)}{\sum_{i=1}^{n} w_{i_{\mathrm{xy}}}}$ $\bar{\mathrm{MD}}=\frac{\sum_{i=1}^{n} w_{i_{\mathrm{xy}}}\cdot\left( y_{i}-x_{i} \right)}{\sum_{i=1}^{n} w_{i_{\mathrm{xy}}}}$

Hereby $w_{i_{hem\_xy}}=1/ (1/w_{i_{xy_{r}}}+1/w_{i_{xy\_l}}$), $w_{i_{xy}}=1/\sigma_{i_{xy}}^{2}$ $w_{i_{\mathrm{xy}}}=1/\sigma_{i_{\mathrm{xy}}}^{2}$ with $\sigma_{i_{xy}}^{2}=\sigma_{i_{x}}^{2}+\sigma_{i_{y}}^{2}+2\cdot Cov(x,y)$, $\sigma_{i_{\mathrm{xy}}}^{2}=$is the combined weighting factor of two sessions that considers the variation in spectra quality, while (*σ*_i_) expresses the absolute SD of the calculated fit, processed by Tarquin (Gordji-Nejad et al. 2018).

The weighted SD_r-l_ of differences (SDD) is calculated as:

${SDD}_{hem}=\sqrt{\frac{\left( n-1 \right)\cdot\sum_{i=1}^{n} w_{i_{hem\_xy}}\cdot\left[ \left( \Delta_{ir}-\Delta_{il} \right)- \bar{{MD}_{hem}} \right]^{2}}{n\cdot\sum_{i=1}^{n} w_{i_{hem\_xy}}}}$

(2)

The weighted hemispheric mean difference $\bar{MD}$*_hem_Cr_Pl_* for a given voxel of *n* (n=15) subjects receiving creatine $\Delta_{i_{hem\_Cr}=}\Delta_{i_{r\_Cr}-}\Delta_{i_{l\_Cr}}$ versus placebo $\Delta_{i_{hem\_Pl}=}\Delta_{i_{r\_Pl}-}\Delta_{i_{l\_Pl}}$ was calculated as:

$\bar{MD}_{hem\_Cr\_Pl}=\frac{\sum_{i=1}^{n} w_{i_{hem\_Cr\_Pl} \cdot}\left[ \Delta_{i_{hem\_Cr}}\cdot({X_{iCr\_r}/X}_{iPl\_r})-\Delta_{i_{hem\_Pl}} \right]}{\sum_{i=1}^{n} w_{i_{hem\_Cr\_Pl}}}$ $\bar{\mathrm{MD}}=\frac{\sum_{i=1}^{n} w_{i_{\mathrm{xy}}}\cdot\left( y_{i}-x_{i} \right)}{\sum_{i=1}^{n} w_{i_{\mathrm{xy}}}}$ , $\bar{\mathrm{MD}}=\frac{\sum_{i=1}^{n} w_{i_{\mathrm{xy}}}\cdot\left( y_{i}-x_{i} \right)}{\sum_{i=1}^{n} w_{i_{\mathrm{xy}}}}$

(3)

(4)

with $w_{i_{hem\_Cr\_Pl}}=1/(\frac{1}{w_{i_{hem\_xy_{Cr}}}}+\frac{1}{w_{i_{hem\_xy_{Pl}}}})$

and the weighted SD of differences (SDD) as:

(5)

$${SDD}_{hem\_Cr-Pl}=\sqrt{\frac{\left( n-1 \right)\cdot\sum_{i=1}^{n} w_{i_{hem\_Cr\_Pl}}\cdot\left[ \Delta_{i_{hem\_Cr}}\cdot({X_{iCr}/X}_{iPl})-\Delta_{i_{hem\_Pl}}\cdot-\bar{MD}_{hem\_Cr\_Pl} \right]^{2}}{n\cdot\sum_{i=1}^{n} w_{{ihem\_}_{Cr\_Pl}}}}$$

The *T* value is then defined by:

(6)

$T=\sqrt{n}\cdot\frac{\bar{MD}_{hem}}{{SDD}_{hem}}$

The corresponding *p* value was calculated using the *t*, *p* conversion table integrated in Microsoft Excel as tvert(abs(*t*); *n*-2;2), with *n*-2 degrees of freedom and two sides (NIST 1996). Grid averages were calculated without weighting, each region was equally accounted for.

Correlations of changes in metabolic and cognitive parameters across n subjects were calculated using Spearman correlation coefficient (r) and the T value defined as:

(7)

$$T= \frac{r \cdot\sqrt{n-2}}{\sqrt{1-r^{2}}}$$

**2.4 Segmentation of WM, GM and CSF**

To understand the composition of grey matter (GM), white matter (WM), and cerebrospinal fluid CSF of the 1H-MRS (PRESS) voxels, 1 mm³ 3D-MPRAGE datasets were segmented using the segment routine in default settings provided in SPM12 (statistical parametric mapping, The Welcome Trust Centre for Neuroimaging).

Regarding CSI, fractions of WM, GM, and CSF were determined using N30R83 atlas-based segmentation of 1 mmT³ 3D-MPRAGE datasets provided by Pmod Neurotool 4.0 (Pmod, Zurich Switzerland). A fixed set of cubic 8 × 8 (25 mm)³ volumes of interest, identical to the CSI grid, was constructed and placed at the exact position displayed by TARQUIN. The desired regional fraction of WM, GM, and CSF was read out. The results, averaged across all subjects, are shown in **Tab.S4**. Signal or signal-ratio changes versus baseline withstanding Bonferroni correction were identified and highlighted in bold script.

**2.5 Signal changes due to shifts of the CSI-grid with repeated positioning of the subjects**

The signal measured in a voxel is considered as the sum of contributions from WM, GM, and CSF where a specific metabolite found in different concentrations. Methodologically most reliable concentration ratios GM/WM found in the literature are in ranges of 1.2 and 1 for PCr and Pi. The ratio CSF to total was ≤ 0.0007 in all reports (Krukowski 2010; W Pan 1998; Zhu 2004; Loreen Ruhm 2021; Hetherington HP. Spencer DD 2001; Dudley 2014; Y Wang 1998; Nukui 2021; Ågren 1988;). The contribution of signal changes for a given voxel due to the spatial shift between a time point (0 pm, 2 am or 4 am) and baseline (6pm) was calculated by the sum in fractional change of ΔWM, ΔGM and ΔCSF multiplied by the concentration ratios of the metabolite in the respective fractions and the respective voxels total signal. Regarding tCr, PCr, and ATP, the maximum of the literature range, namely GM/WM-ratios of 1.87 for tCr, 1.22 for PCr, and 0.69 for ATP were chosen for error estimation.

1. **Results**

**3.1 HEP consumption, hemispheric asymmetry of the response to SD in under placebo**

Measurements revealed a decrease in the right compared to left hemisphere versus baseline in the averaged middle grid (-4.3 ± 1.9%, *p*_13_= .04, *t*_13_ = -2.24) at 2 am and in the upper grid (-9.7 ± 3.8%, *p*_13_= .02, *t*_13_ = -2.59). The decrease was revealed regionally at 4 am in the medial premotor (-9.8 ± 2.7%, *p*_13_= .003, *t*_13_ = -3.59) **(Tab. 2, Fig. 3 A)** region.

At 4 am the decrease in PCr/Pi versus baseline was significantly prominent in the left compared to the right hemisphere in the motor (-15.3 ± 6.1%, *p*_13_= .03, *t*_13_ = -2.49) and posterior F1 (-8.2 ± 3.3%, *p*_13_= .03, *t*_13_ = -2.49) region **(Tab. 2, Fig. 3 A)**. When decreases of 3 time points versus baseline were pooled, additional left-right asymmetries were significant in the thalamus (-4.4 ± 1.6%, *p*_43_= .01, *t*_43_ = -2.52) and posterior F1 region (-4.2 ± 1.8%, *p*_43_= .02, *t*_43_ = -2.38).

Asymmetry of the decrease in ATP-ß/^31^P versus baseline was revealed in medial occipital (-4.1 ± 1.6%, *p*_13_= .02, *t*_13_ = -2.63) and medial premotor (-7.2 ± 2.4%, *p*_13_= .01, *t*_13_ = -2.95) region at 0 pm, in the temporal transversal (-11.8 ± 3.8%, *p*_13_= .01, *t*_13_ = -3.07) and corpus callosum (-4.1 ± 1.7%, *p*_13_= .03, *t*_13_ = -1.7) at 2 am, in the medial occipital (-6.3 ± 2.6%, *p*_13_= .03, *t*_13_ = -2.6) at 4 am and in lateral premotor (-10.6 ± 4.1%, *p*_43_= .01, *t*_43_ = -2.68) and anterior lateral parietal (-11.3 ± 4.2%, *p*_43_= .01, *t*_43_ = -2.46) region, when data of 3 time points were pooled. A decline in ATP-ß/^31^P level versus baseline in the left compared to the right hemisphere significantly differed only at 2 am in the anterior cingulum (-5.2 ± 1.8%, *p*_13_= .01, *t*_13_ = -2.94) **(Tab. 2, Fig. 3 A)**.

**3.2 HEP consumption, hemispheric asymmetry of the response to SD under creatine**

At 0 pm a left hemispheric predominance of the increase in PCr/Pi versus baseline was observed in the posterior superior temporal (-17.4 ± 5.9%, p_13_ = 0.01, t_13_ = -2.94) region. Similar, the left – right difference was significant in the occipito temporal (-17.6 ± 6.8%, p13 = 0.02, t13 = -2.60), and the occipito-temporal (-10.4 ± 4.1%, p_43_ = 0.01, t_43_ = -2.94) region when the changes versus baseline of the 3 time points were pooled. At 4am the left hemisphere showed a regional decrease in the caudate putamen (6.0 ± 2.2%, p_13_ = 0.02, t_13_ = -2.69) (Fig. 2 B).

Regional decrease in ATP-ß/^31^P versus baseline revealed in the thalamus (-4.5 ± 1.7%, *p*_13_= .02, *t*_13_ =2.62). In turn a decrease in the right compared to left hemisphere revealed in insula (-5.1 ± 2.3%, *p*_13_= .05, *t*_13_ = -2.18) at 0pm and in caudatus putamen (-3.6 ± 1.6%, *p*_13_= .04, *t*_13_ = -2.28) at 4am and in insula (-5.7 ± 2.3%, *p*_43_= .01, *t*_43_ = -2.52), temporal transversal (-4.4 ± 1.7%, *p*_43_= .03, *t*_43_ = -2.22) and precuneus (-5.3 ± 2.1%, *p*_43_= .02, *t*_43_ = -2.38) when pooled at 3 time points.

**3.3 HEP consumption, hemispheric asymmetry of the response to creatine versus placebo during SD**

The triple contrast, decrease from baseline at 2 am in ATP-ß/^31^P under creatine versus placebo, left versus right hemisphere revealed a left hemispheric predominance in the temporal transversal region (-10.9 ± 1.6%, *p*_13_= .02, *t*_13_ = -2.65). In turn, in the anterior cingulum a right hemispheric decrease by (8.9 ± 1.6%, *p*_13_= .02, *t*_13_ = -2.59) prevailed.

**Figure S1**


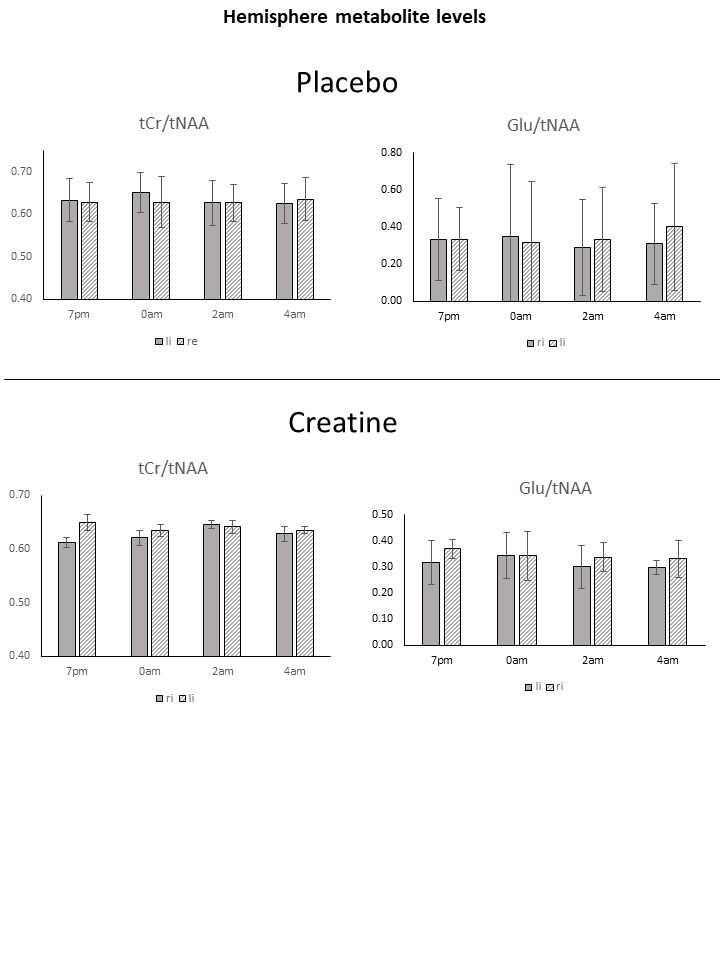


**Fig. S1** tCr/tNAA and Glu/tNAA levels in the medial parietal (25 mm)^3^-single voxels of the left and right hemisphere at measurement time points 6 pm, 0 pm, 2 am and 4 am.

**Figure S2**


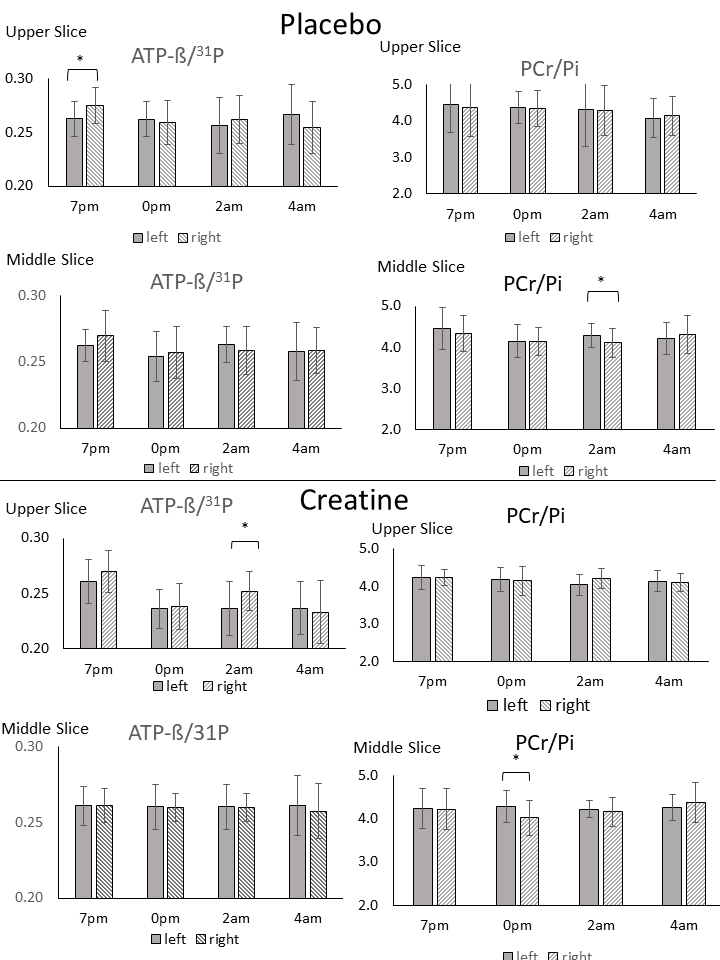


**Fig. S2** ATP-ß/^31^P and PCr/Pi levels averaged across the 9 voxels of the middle grid and the 9 voxels of the upper grid, respectively, in the left and right hemisphere at measurement time points 6 pm, 0 pm, 2 am and 4 am. *, p ≤ 0.05 indicates significant differences.

**Figure S3**


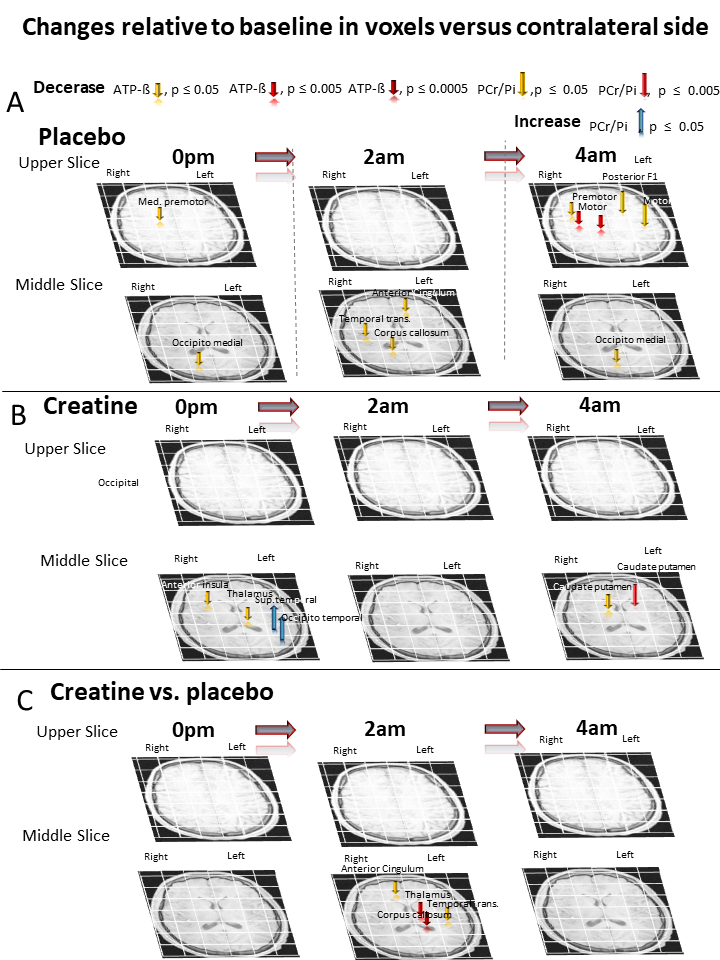


**Fig. S3** Significant changes relative to baseline (6 pm) in PCr/Pi (large arrows) and ATP-ß/^31^P (small arrows) in voxels versus the respective contralateral equivalent after placebo (A) or creatine (B) administration at 0 am, 2 am, and 4 am. Changes in creatine versus placebo are shown in (C). Significance levels are color-coded and indicated by arrows onto axial brain slices in radiological orientation (the right side of the brain is on the left side of the viewer). Those withstanding the Bonferroni and Beniamini Hochberg correction are colored in red and dark red.

**Table S1a** Effect of SD under placebo. Pearson’s correlation coefficients (*r_p,_ p*-value, time point) of the inter-hemispheric differences of changes in PCr/Pi or tCr/tNAA and cognitive scores at 0 pm, 2 am or 4 am under SD versus baseline. Values in bold represent significances that withstand the Bonferroni correction.

| **ΔPCr/Pi**_SD-BL_ | **Middle grid** | **PVT-Speed 0.1Pc,** | **WMT** | **Digit Span** | **Language** | **Logic** | **Numeric** |
| --- | --- | --- | --- | --- | --- | --- | --- |
| Anterior cingulum | R3 (C4-C5) |  | 0.57,._03, 0 am_ |  |  |  |  |
| Anterior insula | R4 (C3-C6) |  | - 0.56,._04, 4am_ |  |  |  |  |
| Caudatus-putamen | R4 (C4-C5) | 0.68,._01, 0 am_ |  |  |  |  |  |
| Capsulo-thalamic | R5 (C4-C5) | 0.56,._04, 0 am_ |  | 0.52,._05, 2 am_ |  |  |  |
| Temporal medulla | R6 (C3-C6) |  |  |  |  |  | 0.68,._01, 2 am_ |
| Corpus Callusum | R6 (C4-C5) |  |  |  |  |  |  |
| Occipito-medial | R7 (C4-C5) |  | - 0.54,._05, 4am_ |  |  |  |  |
| Occipito-temp | R7 (C3-C6) |  |  |  |  |  | 0.53,._04, 2 am_ |
| Mean |  | 0.56,._04_ | - 0.55,._04, 4am_ |  |  |  |  |
| **ΔPCr/Pi**_SD-BL_ | **Upper grid** | **PVT-Speed 0.1Pc,** | **WMT** | **Digit Span** | **Language** | **Logic** | **Numeric** |
| Lateral premotor | R4 (C3-C6) |  |  |  | - 0.65,._01, 0am_  - 0.64,._01, 2am_ |  |  |
| Posterior F1 | R4 (C4-C5) |  |  |  | **- 0.77,._0001, 0am_**  - 0.60,._02, 2am_ |  |  |
| Motor | R5 (C3-C6) |  |  | - 0.52,._05, 2am_ | **- 0.83,._0001, 0am_** |  |  |
| Medial premotor | R5 (C4-C5) |  | 0.55,._04, 0am_ |  | **- 0.78,._001, 0am_**  - 0.52,._05, 2am_ |  |  |
| Anterior-lat. parietal | R6 (C3-C6) |  |  | 0.52,._05, 0am_ |  |  |  |
| Medial central | R6 (C4-C5) |  | 0.69,._01, 2am_ |  |  |  |  |
| Precuneus | R7 (C4-C5) |  |  |  | - 0.53,._04, 0am_ |  |  |
| Postero-lat. parietal | R7 (C3-C6) |  |  |  | - 0.56,._03, 0am_ |  |  |
| Mean |  |  |  |  | **- 0.75,._001, 0am_** |  |  |
| **ΔATP/31P**_SD-BL_ | **Middle grid** | **PVT-Speed 0.1Pc,** | **WMT** | **Digit Span** | **Language** | **Logic** | **Numeric** |
| Anterior cingulum | R3 (C4-C5) |  |  |  |  |  | 0.63,._01, 0am_ |
| Post. sup temporal | R3 (C3-C6) |  |  |  | 0.67,._01, 0am_ |  |  |
| Temporal-transversal | R5 (C3-C6) |  |  |  | 0.65,._01, 2am_ |  |  |
| Temporal medulla | R6 (C3-C6) |  |  | 0.54,._04, 2am_ |  |  |  |
| Occipito-medial | R7 (C4-C5) |  |  | 0.55,._04, 2am_ |  |  |  |
| Occipito-temp | R7 (C3-C6) |  | -0.67,._01, 0am_ | 0.55,._04, 2am_ |  |  |  |
| Mean |  |  |  | 0.55,._04_ |  |  |  |
| **ΔATP/31P**_SD-BL_ | **Upper grid** | **PVT-Speed 0.9Pc,** | **WMT** | **Digit Span** | **Language** | **Logic** | **Numeric** |
| Anterior cingulum | R3 (C4-C5) |  |  |  |  | -0.59,._02, 4am_ |  |
| Caudatus-putamen | R4 (C4-C5) |  |  |  |  | -0.61,._02, 2am_ | 0.59,._02, 4am_ |
| Capsulo-thalamic | R5 (C4-C5) |  |  |  |  | **-0.70,._004, 2am_** |  |

BL, baseline; PVT, psychomotor vigilance test; 0.1 Pc, 10%-percentile of a series of 30-40 trials; 0.9 Pc, 90%-percentile of a series of 30-40 trials; SD, sleep deprivation; WMT, word memory test.

**Table S1b** Effect of SD under creatine. C.f. **Table S7a**.

| **ΔPCr/Pi**_SD-BL_ | **Middle grid** | **PVT-Speed 0.1Pc,** | **WMT** | **Digit Span** | **Language** | **Logic** | **Numeric** |
| --- | --- | --- | --- | --- | --- | --- | --- |
| Anterior cingulum | R3 (C4-C5) |  | -0.58,._03, 2am_ |  |  |  | -0.59,._02, 0am_ |
| Post. sup temporal | R3 (C3-C6) |  | 0.65,._01, 2am_ |  |  |  |  |
| Anterior insula | R4 (C3-C6) |  |  |  |  |  | 0.59,._02, 2am_ |
| Caudatus-putamen | R4 (C4-C5) |  |  | 0.68,._01, 4am_ |  |  |  |
| Temporal-transversal | R5 (C3-C6) |  |  | 0.59,._02, 0am_ | 0.51,._05, 4am_ | 0.53,._04, 4am_ |  |
| Temporal medulla | R6 (C3-C6) |  | -0.64,._01, 2am_ | 0.59,._03, 0am_  0.56,._04, 4am_ |  |  |  |
| Corpus Callusum | R6 (C4-C5) |  | -0.55,._04, 0am_  -0.64,._01, 2am_ | 0.60,._02, 0am_ |  |  |  |
| Occipito-medial | R7 (C4-C5) | -0.59,._03, 0am_ | -0.63,._01, 2am_ |  |  |  |  |
| Mean |  |  | -0.59,._03_ | 0.63,._02_ |  |  |  |
| **ΔPCr/Pi**_SD-BL_ | **Upper grid** | **PVT-Speed 0.1Pc,** | **WMT** | **Digit Span** | **Language** | **Logic** | **Numeric** |
| Precuneus | R7 (C4-C5) |  |  |  | -0.57,._03, 0am_  0.52,._05, 2am_ |  |  |
| Postero-lat. parietal | R7 (C3-C6) |  |  | 0.60,._02, 4am_ |  |  |  |
| **ΔATP/31P**_SD-BL_ | **Middle grid** | **PVT-Speed 0.1Pc,** | **WMT** | **Digit Span** | **Language** | **Logic** | **Numeric** |
| Anterior insula | R4 (C3-C6) |  | -0.61,._02, 0am_ |  |  |  |  |
| Caudatus-putamen | R4 (C4-C5) | -0.63,._02, 2am_ |  |  |  |  |  |
| Temporal medulla | R6 (C3-C6) | 0.61,._02, 0am_ |  | 0.54,._05, 4am_ |  |  |  |
| Occipito-medial | R7 (C4-C5) |  |  |  |  |  | -0.58,._02, 4am_ |
| **ΔATP/31P**_SD-BL_ | **Upper grid** | **PVT-Speed 0.1Pc,** | **WMT** | **Digit Span** | **Language** | **Logic** | **Numeric** |
| Anterior F1 | R3 (C4-C5) |  |  | -0.62,._02,0am_ | -0.52,._04,0am_ | 0.66,._01,4am_ |  |
| Posterior F1 | R4 (C4-C5) |  | 0.59,._03,0am_ |  |  |  |  |
| Motor | R5 (C3-C6) |  |  |  |  | 0.61,._01,0am_  0.54,._04,4am_ |  |
| Precuneus | R7 (C4-C5) | -0.68,._01, 4am_ |  |  |  | -0.60,._02,4am_ |  |

**Table S1c** The same as Table S7a, but data from time points 0 am, 2 am and 4 am were pooled.

| **ΔPCr/Pi**_SD-BL_ | **Middle grid** | **PVT-Speed 0.1Pc,** | **WMT** | **Digit Span** | **Language** | **Logic** | **Numeric** |
| --- | --- | --- | --- | --- | --- | --- | --- |
| Thalamo-capsular | R5 (C4-C5) | 0.41,2.98,._005_ |  |  |  |  |  |
| **ΔPCr/Pi**_SD-BL_ | **Upper grid** | **PVT-Speed 0.1Pc,** | **WMT** | **Digit Span** | **Language** | **Logic** | **Numeric** |
| Lateral premotor | R4 (C3-C6) |  |  |  | -0.59,4.79,._00002_ |  |  |
| Posterior F1 | R4 (C4-C5) |  |  |  | -0.56,4.39,._0001_ |  |  |
| Motor | R5 (C3-C6) |  | 0.49,3.62,._001_ |  | -0.49,3.64,._001_ |  |  |
| Medial premotor | R5 (C4-C5) |  | 0.48,3.50,._0001_ |  | -0.39,2.81,._007_ |  |  |
| Medial central | R6 (C4-C5) |  | 0.43,3.02,._004_ |  | -0.42,3.02,._004_ |  |  |
| Postero-lat. parietal | R7 (C3-C6) |  |  |  |  | 0.50,3.74,._0005_ |  |
| Mean |  |  |  |  | -0.52,3.97,._0003_ |  |  |
| **ΔATP/31P**_SD-BL_ | **Middle grid** | **PVT-Speed 0.1Pc,** | **WMT** | **Digit Span** | **Language** | **Logic** | **Numeric** |
| Occipito-temp | R7 (C3-C6) |  | -0.48,3.49,._001_ |  |  |  |  |
| **ΔATP/31P**_SD-BL_ | **Upper grid** | **PVT-Speed 0.9Pc,** | **WMT** | **Digit Span** | **Language** | **Logic** | **Numeric** |
| Medial premotor | R5 (C4-C5) |  |  |  |  | -0.50,3.72,._0006_ |  |
| Posterior F1 | R4 (C4-C5) |  |  |  |  | -0.48,3.48,._001_ |  |

**Table S1 d** The same as Table S7b, but data from time points 0 am, 2 am and 4 am were pooled.

| **ΔPCr/Pi**_SD-BL_ | **Upper grid** | **PVT-Speed 0.1Pc,** | **WMT** | **Digit Span** | **Language** | **Logic** | **Numeric** |
| --- | --- | --- | --- | --- | --- | --- | --- |
| Lateral premotor | R4 (C3-C6) |  | 0.53,4.06,._0002_ |  |  |  |  |
| Motor | R5 (C3-C6) |  | 0.45,3.28,._002_ |  |  |  |  |
| **ΔATP/31P**_SD-BL_ | **Middle grid** | **PVT-Speed 0.1Pc,** | **WMT** | **Digit Span** | **Language** | **Logic** | **Numeric** |
| Anterior insula | R4 (C3-C6) |  | -0.44,3.25,._002_ |  |  |  |  |
| **ΔATP/31P**_SD-BL_ | **Upper grid** | **PVT-Speed 0.1Pc,** | **WMT** | **Digit Span** | **Language** | **Logic** | **Numeric** |
| Precuneus | R7 (C4-C5) |  |  | -0.49,3.66,._001_ |  |  |  |
| Motor | R5 (C3-C6) |  |  |  |  | 0.50,3.82,._0004_ |  |

**Table S1 e** Pearson’s correlation coefficients of the inter-hemispheric differences of changes in ATP-ß and score in KKS and FAT withstanding the Bonferroni correction from time points 0 am, 2 am and 4 am were pooled.

| **ΔATP/31P**_SD-BL_ | **Middle grid** | **FAT** | **KSS** |
| --- | --- | --- | --- |
| Temporal medulla | R6 (C3-C6) |  | 0.54,4.19,._0001_ |
| Occipito-temp | R7 (C3-C6) |  | 0.55,4.35,._0001_ |
| Corpus Callusum | R6 (C4-C5) | 0.42,2.99,._005_ |  |
| Capsulo-thalamic | R5 (C4-C5) | 0.43,3.12,._003_ |  |
| Mean |  |  | 0.49,3.73,._001_ |
| **ΔATP/31P**_SD-BL_ | **Upper grid** |  |  |
| Precuneus | R7 (C4-C5) |  | 0.43,3.09,._004_ |

**Table S2**

Estimate of errors induced by de –novo positioning of the subjects at each measurement run. Expected mean within-subject changes in PCr/Pi signals induced by spatial shift of the grid position between baseline (6 pm) and 0 pm, 2 am and 4 am. Calculated were signal-changes which could be expected assuming a fixed ratio of the ^31^P-MRS-Signals from GM and WM and zero contribution of CSF. Assumed ratios were 1.20 for PCr and 1 for Pi. Voxels with significant changes withstanding the Bonferroni correction are highlighted in **bold** characters.

| **Middle Slice** | | | | **Δ (PCr/Pi)** | | | | | |
| --- | --- | --- | --- | --- | --- | --- | --- | --- | --- |
| Voxel No | Hs | Anatomical label | | **0 pm vs. 6 pm** | | **2am vs. 6 pm** | | **4 am vs. 6 pm** | |
|  |  |  |  | **Placebo** | **Creat.** | **Placebo** | **Creat.** | **Placebo** | **Creat.** |
| R5C3 | r | Temporal transversal | | -0.05% | -0.1% | -0.35% | -0.1% | -0.08% | -0.9% |
| R5C6 | l |  |  | -0.13% | -0.1% | -0.40% | -0.1% | -0.43% | 0.2% |
| R3C4 | r | Anterior cingulum | | 0.20% | 0.1% | -0.12% | -0.1% | -0.06% | -0.1% |
| R3C5 | l |  |  | -0.30% | -0.2% | -0.29% | -0.2% | 0.05% | 0.0% |
| R4C4 | r | Capsulo-striatal | | -0.21% | 0.3% | 0.08% | 0.2% | -0.07% | -0.4% |
| R4C5 | l |  |  | 0.13% | 0.1% | -0.06% | -0.3% | -0.27% | -1.0% |
| R5C4 | r | Thalamo-capsular | | -0.47% | -0.1% | -0.07% | 0.1% | -0.27% | -0.7% |
| R5C5 | l |  |  | -0.16% | **0.7%** | 0.46% | 0.4% | -0.01% | -0.4% |
| R6C4 | r | Corpus callosum | | -0.20% | 0.0% | -0.12% | -0.4% | -0.31% | 0.0% |
| R6C5 | l |  |  | 0.27% | -0.7% | 0.33% | -0.8% | -0.10% | -0.7% |
| R7C4 | r | Occipito-medial | | -0.28% | -0.1% | -0.25% | -0.1% | -0.76% | 0.0% |
| R7C5 | l |  |  | 0.18% | -0.2% | 0.03% | 0.0% | -0.61% | -0.5% |
| **Upper Slice** | | | | **0 pm vs. 6 pm** | | **2am vs. 6 pm** | | **4 am vs. 6 pm** | |
|  |  |  |  | **Placebo** | **Creat.** | **Placebo** | **Creat.** | **Placebo** | **Creat.** |
| R4C3 | r | | Lateral  premotor | -0.15% | -1.3% | -0.06% | 0.2% | -0.10% | -0.8% |
| R4C6 | l | |  | 0.26% | -1.2% | 0.49% | 0.0% | -0.38% | -0.8% |
| R5C3 | r | | Motor | -0.46% | -0.3% | -0.09% | -0.4% | -0.15% | -0.3% |
| R5C6 | l | |  | -0.15% | 0.2% | -0.01% | 0.0% | -0.39% | -0.2% |
| R6C3 | r | | Ant. later. parietal | -0.56% | -0.1% | -0.74% | 0.0% | -0.82% | -0.5% |
| R6C6 | l | |  | -0.14% | -0.2% | 0.20% | 0.2% | -0.45% | -0.3% |
| R7C3 | r | | Post. lateral parietal | -1.61% | -0.7% | -0.79% | -0.7% | -0.26% | 0.4% |
| R7C6 | l | |  | -0.28% | -1.0% | -0.50% | -0.2% | -1.49% | 0.8% |
| R4C4 | r | | Posterior F1 | 0.03% | -0.4% | -0.63% | -0.1% | -0.05% | 0.0% |
| R4C5 | l | |  | 0.62% | -0.1% | 0.59% | -0.2% | 0.48% | -0.3% |
| R5C4 | r | | Medial  premotor | -0.26% | -0.6% | -0.55% | 0.0% | -0.22% | -0.2% |
| R5C5 | l | |  | 0.30% | 0.5% | -0.07% | -0.1% | 0.11% | 0.2% |

**Table S3**

Spatial shift of the grid position between time points.

| **Spatial shift of the CSI-grid (mm)** | | | | | | | | | | | | | |
| --- | --- | --- | --- | --- | --- | --- | --- | --- | --- | --- | --- | --- | --- |
| Middle slice | 0 pm vs. 6 pm | | 2am vs. 6 pm | | 4 am vs. 6 pm | | Upper  slice | 0 pm vs. 6 pm | | 2am vs. 6 pm | | 4 am vs. 6 pm | |
|  | **Placebo** | **Creat.** | **Placebo** | **Creat.** | **Placebo** | **Creat.** |  | **Placebo** | **Creat.** | **Placebo** | **Creat.** | **Placebo** | **Creat.** |
| Δx | 0.9 | 1.0 | 1.0 | 0.6 | 1.1 | 0.8 | Δx | -0.2 | 0.6 | 0.3 | 0.5 | 1.0 | 0.8 |
| Δy | 1.2 | 0.7 | -0.7 | 0.5 | 0.7 | 1.1 | Δy | 1.1 | -0.3 | 1.3 | 1.1 | 1.3 | -0.6 |
| Δz | -0.1 | 1.1 | 0.8 | -0.3 | 1.4 | 0.5 | Δz | 0.5 | 1.2 | 0.6 | 0.2 | 0.7 | 0.7 |

**Table S4**

Fractions of WM, GM and CSF in 1H PRESS voxels and in CSI with significant changes withstanding the Bonferroni correction extracted from segments of co-registered MPRAGE datasets, averaged across all subjects.

| **Middle Slice** | | | | | | |
| --- | --- | --- | --- | --- | --- | --- |
| Voxel No | Hs | Anatomical label | | **WM** | **GM** | **CSF** |
| R5C3 | r | Temporal transversal | | 0.35 | 0.57 | 0.09 |
| R5C6 | l |  |  | 0.32 | 0.58 | 0.10 |
| R3C4 | r | Anterior cingulum | | 0.55 | 0.40 | 0.05 |
| R3C5 | l |  |  | 0.45 | 0.46 | 0.08 |
| R4C4 | r | Capsulo-striatal | | 0.37 | 0.48 | 0.15 |
| R4C5 | l |  |  | 0.33 | 0.44 | 0.21 |
| R5C4 | r | Thalamo-capsular | | 0.39 | 0.50 | 0.11 |
| R5C5 | l |  |  | 0.36 | 0.50 | 0.15 |
| R6C4 | r | Corpus callosum | | 0.40 | 0.29 | 0.30 |
| R6C5 | l |  |  | 0.38 | 0.28 | 0.33 |
| R7C4 | r | Occipito-medial | | 0.37 | 0.55 | 0.08 |
| R7C5 | l |  |  | 0.32 | 0.55 | 0.12 |
| **Upper Slice** | | | | | | |
| Voxel No | Hs | | Anatomical label | **WM** | **GM** | **CSF** |
| R4C3 | r | | Lateral  premotor | 0.25 | 0.64 | 0.11 |
| R4C6 | l | |  | 0.33 | 0.59 | 0.08 |
| R5C3 | r | | Motor | 0.47 | 0.44 | 0.08 |
| R5C6 | l | |  | 0.52 | 0.42 | 0.07 |
| R6C3 | r | | Ant. later. parietal | 0.40 | 0.48 | 0.12 |
| R6C6 | l | |  | 0.45 | 0.44 | 0.11 |
| R7C3 | r | | Post. lateral parietal | 0.17 | 0.70 | 0.14 |
| R7C6 | l | |  | 0.20 | 0.65 | 0.15 |
| R4C4 | r | | Posterior F1 | 0.54 | 0.39 | 0.07 |
| R4C5 | l | |  | 0.48 | 0.38 | 0.13 |
| R5C4 | r | | Medial  premotor | 0.64 | 0.30 | 0.06 |
| R5C5 | l | |  | 0.61 | 0.31 | 0.09 |
|  | | | | | | |
| **Anatomical label** | | | | **WM** | **GM** | **CSF** |
| r | | | Ant. med. parietal | 0.61 | 0.32 | 0.06 |
| l | | |  | 0.60 | 0.34 | 0.06 |
